# Prenatal maternal life adversity impacts on learning and memory in offspring: implication to transgenerational epigenetic inheritance

**DOI:** 10.3389/fnins.2025.1518046

**Published:** 2025-02-13

**Authors:** Prince David Adeline Dorothy, Koilmani Emmanuvel Rajan

**Affiliations:** Behavioural Neuroscience Laboratory, Department of Animal Science, Bharathidasan University, Tiruchirappalli, India

**Keywords:** prenatal maternal stress, working memory, DNA methylation, corticosterone, oxytocin, brain-derived neurotrophic factor, transgenerational inheritance, epigenetic mechanism

## Abstract

Maternal stress exposure during pregnancy is known to affect offspring behavior, including learning and memory. We hypothesized that maternal stress-induced changes transmit this effect through maternal line mediated transgenerational epigenetic inheritance. To test our hypothesis, pregnant rats (F0) were undisturbed (Control, Ctrl)/exposed to social stress during gestational days (GD) 16–18 (PMS)/exposed to social stress and treated with oxytocin during GD-16 to 18 (PMS+OXT). Subsequently, F1 female offspring from Ctrl, PMS, and PMS+OXT were mated with Ctrl F1 males to examine maternal line mediated transgenerational impacts. Female animals (F1 and F2) were subjected to behavioral test and the levels of global H3K4me2/H3K4me3 methylation, methylation in the CRH promoter, expression of *Crh, Crh* receptors (*Crhr1, Crhr2*), and BDNF were determined. It was found that prenatal maternal stress (PMS) reduced reference and working memory in F1 and F2 offspring, increased global and specific H3K4me2, H3K4me3 methylation in the CRH promoter, expression of *Crh, Crh* receptors, and corticosterone (CORT), and down-regulated the expression of pro-and mature BDNF by differentially regulating *Bdnf* transcripts III, IV and VI in the amygdala. Oxytocin exposure reduced PMS-induced global and specific H3K4me2/3 changes, which repressed the expression of *Crh, Crh* receptors, reduced CORT levels, up-regulated the expression of pro-BDNF and mature BDNF, and improved memory in F1 and F2 offspring. Collectively, our study revealed that PMS reduced reference and working memory performance in F1 and F2 offspring through maternal line transgenerational inheritance of H3K4me2, H3K4me3 methylation, and associated mechanisms that regulate BDNF expression and synaptic plasticity.

## 1 Introduction

Clinical and animal studies have demonstrated that physical and social stressors during gestation can generate developmental behavioral disorders in offspring (Coussons-Read, 2013; Dieckmann and Czamara, 2024; Dong and Pandey, 2021; McDonald et al., 2023; Blaze and Roth, 2015). Maternal stress is known to increase corticosterone (CORT) and adrenocorticotrophin (ACTH) levels in maternal plasma (Herman and Cullinan, 1997) as a result of the stress response of hypothalamic—pituitary—adrenal (HPA) axis (Bagley et al., 2019). The maternal placental—fetal (MPF) pathway may not completely attenuate CORT transfer (Kassotaki et al., 2021), which generates a shift in offspring HPA axis homeostasis and alters the developmental trajectory and behavioral phenotype (Babenko et al., 2015). In the HPA axis, corticotrophin-releasing hormone (CRH) acts as the chief mediator of the stress-responsive mechanism to facilitate the release of CORT (Chaves et al., 2021).

CRH exerts its action through its high-affinity G-protein-coupled receptor (GPCR), *Crhr1* and *Crhr2* (Holsboer and Ising, 2008; Inda et al., 2017). Elevated levels of CRH activate its receptors, which alter the transmission of serotonin (5-Hydroxytriptomain, 5-HT), dopamine (DA), and oxytocin (OXT) through a feedback mechanism, and may lead to developmental behavioral changes (Smith and Wang, 2014; Roque et al., 2022; Alcántara-Alonso et al., 2024). Oxytocin (OXT), a peptide (9 aa) hormone synthesized in hypothalamus (paraventricular nuclei, PVN; supraoptic nuclei, SON) (Buijs, 1978), is delivered through the posterior pituitary to the central nervous system (CNS), and other structure in the brain including hippocampus, amygdala, bed nucleus of the stria terminalis (BNST) and nucleus accumbens (NAc) (Knobloch et al., 2012). OXT exerts its function through typical class I G-protein receptor (GPCR) (Gimpl and Fahrenholz, 2001) and activates neuron throughout the brain, including in central nucleus of amygdala (CeA) (Lee et al., 2005). OXT acts on the HPA axis *via* negative feedback regulation of CRH and the interaction between CRH and central OXT critically regulates the neuroendocrine response to stress (Winter and Jurek, 2019). CRH inhibits OXT action, and OXT suppresses the stress-mediated induction of CRH expression to maintain homeostasis (Nomura et al., 2003; Janeček and Dabrowska, 2019). Activation of the oxytocin system regulates emotion, stress response in virgin, pregnant and lactating rats (Neumann et al., 2000), social behavior (Neumann, 2008), and fear response (Viviani et al., 2011), and protects memory in stressed rats (Lee et al., 2015). Earlier studies have demonstrated that stressful experiences alter the epigenetic signatures embedded in the genome, specifically genes associated with the HPA axis and stress response (Cánepa and Berardino, 2024; Ohi et al., 2024).

Evidence indicates that maternal stress can alter fetal epigenetic signatures, including methylation of genes implicated in the stress response (Darnaudéry and Maccari, 2008; Dion et al., 2022), and may have a long-lasting impact by potentially inheriting to the following generations (Wiley et al., 2023; Moelling, 2024). Stress has been known to alter Histon-3 Lysine4 (H3K4me2/me3) methylation in the CRH promoter, which facilitates the transcriptional activation of downstream signaling molecules (Hunter et al., 2009). Transcriptional activation of the CRH promoter is often a core factor in the regulation of stress response/resilience, synaptic plasticity, learning, and memory (Fuge et al., 2014). Elevated levels of CRH negatively influence the expression of brain-derived neurotrophic factor (BDNF), and vice versa (Jeanneteau et al., 2012; Hu et al., 2020). BDNF is encoded by multiple bipartite transcripts (exon I-XI), which tightly regulate the expression of BDNF in response to stress response (Colliva and Tongiorgi, 2021) and synaptic plasticity, learning, and memory (Bruijniks et al., 2020; Kim et al., 2024).

A large number of animal and clinical studies have reported the underlying mechanism of the parental effect on transgenerational inheritance based on paternal lineage (reviewed in Dion et al., 2022). We previously reported that prenatal maternal stress (PMS) alters neurobehavior in offspring through stress hormones, neurotransmitters, oxidative stress, and other signaling molecules (Sivasangari and Rajan, 2020; Sivasangari et al., 2023). Several animal models have reported that maternal stress induces long – term effects on offspring, likely through epigenetic changes (Blaze and Roth, 2015; Santilli and Boskovic, 2023), however, there are differences in the types of stressors and markers tested. Therefore, we predicted that maternal line-mediated transgenerational epigenetic inheritance might transmit alterations and behavioral phenotype from F1 to F2 offspring. To test our hypothesis, we designed the following experiment : (i) pregnant rats (F0) were undisturbed (Control, Ctrl), (ii) pregnant rats (F0) were exposed to social stress during gestational day (GD) 16-18 (PMS), and (iii) pregnant rats (F0) were exposed to social stress during GD 16-18 and treated with oxytocin (PMS+OXT) on GD-18. Further F1 female offspring (CtrlF1, PMSF1, PMS+OXTF1) were mated with Ctrl F1males to examine maternal line transgenerational effects.

## 2 Materials and methods

### 2.1 Animal and experimental design

**FIGURE 1:**
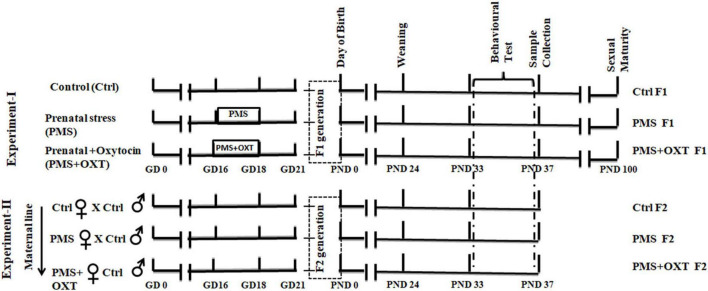
Experimental timeline showing the complete sequence of experiments performed. In this study, two experiments were performed; Control (Ctrl) pregnant rats were housed in standard laboratory condition without any disturbance. Prenatal maternal stress (PMS) provided during gestational day (GD-16 to 18), to stressed mother (F0) –St and stressed mother treated with oxytocin (OXT) during GD-16 to 18, 30 min after stress. F1 offspring (CtrlF1, PMS F1, PMS+OXT F1) were subjected to behavioral test during their early adolescent age (postnatal day -33 to 37). In the second experiment, female F1 offspring (CtrlF1, PMS F1, PMS+OXT F1) were mated with CtrlF1 male, and F2 offspring (CtrlF2, PMSF2, PMS+OXT F2) were subjected to behavioral test.

The estrous cycle of female Wistar rats (*Rattus norvegicus*) was continuously monitored, and selected females were housed individually (43 × 27 × 15 cm) with a male for mating. Rats were maintained under standard laboratory conditions (12 h light/ dark cycle; 22–25°C) with *ad libitum* access to feed (chow pellets) and water. Paddy husks were provided as bedding, and a portion of husk was removed every day to clean and maintain the home ’se odor to avoid stress (Carbone et al., 2016). The day of presence of sperm in the vaginal lavage was noted as gestational day (GD-0; *n* = 19), and pregnant female rats were housed individually. Pregnant female rats were randomly assigned to three groups: (i) control (Ctrl), (ii) prenatal maternal stress (PMS), and (iii) prenatal maternal stress + drug (oxytocin) treatment (PMS + OXT). Pregnant rats in the PMS and PMS+OXT groups were subjected to social stress during GD-16 to 18, and the PMS+OXT group received Oxytocin [0.5 IU (Cat # 103H05241, Sigma Aldrich) in 50μl of PBS intraperitoneally (i.p)]. The dosage of oxytocin was established based on earlier reports, which showed that oxytocin reaches the brain and elicit substantial effects, i.e., memory (Balmus et al., 2018; Pãdurariu et al., 2018). Animals received oxytocin during light-phase (between 9.00 and 11.00) of GD-16-18, 30 min after social stress (Gulevich et al., 2019). F1 female offspring Ctrl F1 (*n* = 13), PMS F1 (*n* = 15), and PMS+OXT F1 (*n* = 17) were subjected to behavioral test during their early adolescent postnatal day (PND) 32–36, and samples were processed and retained for the F2 experiment. In the second set of experiments, sexually matured adult F1 females from the CtrlF1 (*n* = 7), PMS F1 (*n* = 8), and PMS+OXT F1 (*n* = 8) groups were selected randomly, housed individually, and allowed to mate with CtrlF1 male (never exposed to stress). F2 offspring from control (CtrlF2; *n* = 8), stressed (PMS F2, *n* = 11), stressed, and oxytocin treated (PMS+OXT F2; *n* = 8) groups were subjected to behavioral testing and samples were processed for molecular analysis (Figure 1). Animals were treated following guidelines of Committee for the Purpose of Control and Supervision of Experiments on Animals (CPCSEA, India) and all the protocols are approved (BDU/IAEC/P23/2022 dated 25.08.2022) by Institutional Animal Ethical Committee (IAEC), Bharathidasan University.

### 2.2 Prenatal maternal stress

Prenatal maternal stress (PMS) was induced individually by allowing the pregnant rat to interact with a resident stranger (senescent male; 24 months old) from GD-16 to GD-18 (10 min/day), as previously reported earlier (Sivasangari and Rajan, 2020; Sivasangari et al., 2023). A three chambers social defeat apparatus [30 × 30 × 30 cm each, resident chamber (RC), intruder chamber (IC), and observer chamber (OC)] was connected to a housing laboratory cage (43 × 27 × 15 cm) for resident stranger (Supplementary Figure S1). Pregnant rats were introduced into the OC, and after five minutes the interaction was facilitated by opening the sliding wire mesh door, the pregnant rats were allowed to interact with the stranger (10 min/day). The control rats were allowed to explore the clean apparatus in the absence of a stranger.

### 2.3 Behavioral test

#### 2.3.1 Hole—board test

Hole-board test (HBT) apparatus (44.5 × 44.5 × 30.5 cm) was originally designed with 16 holes, i.e., four identical rows each containing four holes at equidistant (Boissier et al., 1964), and offers to measure various aspects of behavior such as exploration and anxiety, habituation to a novel environment, spatial learning and memory (working and reference memory) (Lundberg et al., 2019, 2020). Therefore, HBT was used to measure the working and reference memory performance. On postnatal day (PND)–33, animals were transferred to the experimental room, after 1 h allowed exploring the apparatus for 10 min. PND-34 animals were food deprived for 8 h and habituated to the apparatus with all holes baited. On PND-35, 36 animals were trained individually; four holes were baited in a fixed pattern, and the other holes were closed. On PND-37, the animals were tested in the HBT by placing baits in four holes in a fixed pattern, and the remaining holes were kept open. The apparatus was wiped with 75% ethanol after each training/testing session to avoid olfactory cues, and all activities were recorded. Ratio of reference memory = no. of visits + revisits to baited holes/Total No. of holes visits. Ratio of working memory = no. of food-rewarded visitors/no. of visits and revisits to baited holes were calculated (Kuc et al., 2006). The time spent freezing in the hole-board apparatus was scored as the absence of all movement except respiration and considered anxiety-like behavior (Treit and Fundytus, 1988).

### 2.4 Sample preparation

Immediately after the behavioral test, female rats [CtrlF1 (*n* = 6), PMS F1 (*n* = 6), PMS+OXT F1 (*n* = 6), CtrlF2 (*n* = 6), PMS F2 (*n* = 6), PMS+OXT F2 (*n* = 6)] were sacrificed, the whole brain was carefully removed and placed on an ice-cold Petri dish, and the amygdala was dissected (Zapala et al., 2005). Tissue (left and right amygdala) samples were divided for preparation of the corticosterone analysis, genomic DNA, total RNA, and total and histone proteins.

#### 2.4.1 Corticosterone analysis

Amygdala tissues (25 mg) were washed in buffer, homogenized (50 μL), and centrifuged (10,000 × g, 4°C, 10 min). Supernatants were collected, and the level of corticosterone (CORT) was estimated using Enzyme-Linked Immunosorbent Assay (ELISA) (ALPCO Diagnostics, Salem, NH) according to the manufacturer’s instructions. The CORT level were calculated in ng/mg for wet tissues.

#### 2.4.2 Western blot analysis

Three samples from each experimental group were pooled together into two groups, and then total protein and histone protein were isolated from each group. Total protein was prepared by homogenize the tissue sample in ice-cold lysis buffer containing protease inhibitor (4 μL/mL) (Sigma-Aldrich, India). The homogenate was incubated on ice (30 min) followed by centrifugation at 4°C (10,000 × *g* for 30 min at 4). The supernatant was collected and centrifuged again at 4°C (12,000 × *g*, 15 min), and aliquots were stored at −80°C.

##### 2.4.2.1 Histone protein

Tissue was homogenized with TX buffer (Tris—HCL, NaCl, EDTA, Triton 100, protease inhibitor cocktail). The homogenates were incubated on ice for 15 min and then centrifuged at 4°C (10 min). The pellet was dissolved in HCL—TX buffer (0.2M) and incubated on ice for 30 min, followed by centrifugation at 4°C (10 min). The supernatant was collected and stored at −80°C.

Samples were quantified (Bio-Rad Protein Assay Kit, Bio-Rad Laboratories Inc., United States) using a Biophotometer Plus (Eppendorf Inc., Germany), and equal concentrations of total protein (50μg) were separated on a polyacrylamide gel (10%). The separated proteins were transferred onto a membrane using a Trans-Blot Turbo Transfer System (Bio-Rad Laboratories, Inc., United States). The membrane was incubated for 2 h in a blocking solution (Tris-buffered saline, Tween-20; TBS-T, 0.1%; non-fat milk, 5%). Membranes were gently washed with TBS-T (5 min/wash) and then incubated in any one of the following primary antibodies [anti-histone H3 rabbit monoclonal antibody (Cat# 4499, 1:5,000; Cell Signalling Technology, Inc.); anti-H3K4me2 rabbit monoclonal antibody (Cat# 9725, 1:5,000; Cell Signalling Technology, Inc.); antiH3K4me3 rabbit monoclonal antibody (Cat# 9725, 1:5,000; Cell Signalling Technology, Inc.); anti-pro-BDNF rabbit polyclonal antibody (Cat# SC-546, 1:2,000; Santa Cruz Biotechnology), mature BDNF rabbit polyclonal antibody (Cat# PA5-85730, 1: 2,000; Invitrogen), and antiß-actin rabbit polyclonal antibody (Cat# SC-47778, 1:2,000; Santa Cruz Biotechnology, Inc.) for about 12–16 h at 4°C. The membrane was washed three times (5 min) in 1XTBS-T and specific protein-bound antibodies were detected by goat anti-rabbit (GeNei^*TM*^, 1:5,000; Cat# 621100180011730)/anti-mouse (GeNei^*TM*^, 1:5,000; Cat# 105215) IgG secondary antibody conjugated with alkaline phosphatase (ALP) by incubating for 4 h at room temperature. Subsequently, the membrane was washed with 1XTBS-T (2 × 5 min), and membrane-bound ALP activity was determined using an alkaline phosphatase substrate (AP Detection Reagent Kit, Merck). After standardizing the western blot using combined linear range detection, the experiment was performed separately for each group. The values from each blot were measured (before saturation) three times in a linear range during development. Images were obtained and the specific band intensity was calculated using a molecular imager (ChemiDoc XRS; Image Lab-2, Bio-Rad Laboratories, Inc., United States). Data showing normalized values with Total H3 / beta-actin, and all uncropped western blot images are shown in Supplementary Information.

#### 2.4.3 Chromatin immunoprecipitation assay

Genomic DNA was isolated from amygdala tissue following the manufacturer’s instructions (Cat# FATGEM-001B; Tissue Genomic DNA extraction kit, Favorgen). The concentration of the DNA sample was estimated using a Biophotometer Plus (Eppendorf Inc., Germany) and stored at −80°C. In the Chromatin Immunoprecipitation (ChIP) assay, immunoprecipitation (IP) was facilitated by incubating genomic DNA (10 μg) with specific antibodies [anti-H3K4me2 rabbit monoclonal antibody/ anti-H3K4me3 rabbit monoclonal antibody/ anti-histone H3 rabbit monoclonal antibody)]. For 12 h at 4°C, the procedure described by the manufacturer (SimpleChIP^®^ Enzymatic Chromatin IP Kit (Magnetic Beads) Cat # 9003. Cell Signalling Technology). Protein G magnetic beads (30 μL) were added to each IP reaction mixture and incubated for 2 h at 4°C in a rotating shaker. The DNA-protein complex was washed with a graded washing solution, and DNA was eluted from the antibody/ protein G magnetic beads using ChIP Elution Buffer. Chromatin was reverse cross-linked by incubating NaCl (5M) and Proteinase K at 65°C for 2 h. DNA was eluted using a DNA spin column following a series of procedures, and the column was washed with DNA binding buffer (centrifuged at 18,500 × g for 30 s), transferred to the sample (450 μL), centrifuged at 18,500 × g for 30 s, and washed with wash buffer by centrifugation at 18,500 × g for 30 s. Finally, the DNA was eluted with DNA elution buffer by centrifugation at 18,500 × g for 30 s, quantified, and stored at -20°C.

Methylation of the CRH promoter was quantified by quantitative real—time chain reaction (qRT–PCR). The total reaction volume (20 μL) contained real-time mixture (SYBR green super mix, Bio-Rad Laboratories Inc.) and specific primers (For 5′CTGTCAAGAGAGCGTCAGC TTATTA-3′ and Rev 5′-CTCTTCAGTTTCTCAAGGTAC TTGGC-3′ each 100 pm) (Singh-Taylor et al., 2018) with the following reaction conditions [denaturation (92°C, 3 min), 40 cycles of denaturation (92°C, 5 s), annealing (63.6°C, 5 s) extension (72°C, 5 s), and final extension (72°C, 10 min). Specific amplification was confirmed by dissociation and melting curve analysis (CFX-96 Touch Real-Time PCR detection system; CFX Manager version 2 software; Bio-Rad Laboratories Inc., United States). The data were normalized to the internal control and presented as the mean fold change.

#### 2.4.4 Quantitative real—time PCR

RNA was isolated using PureZOL (cat. # 732-6880; Bio-Rad Laboratories Inc., United States) and stored at −80°C. Total RNA (2 μg) was used to synthesize cDNA (cat# 170-8891; Iscript™ cDNA synthesis kit, Bio-Rad Laboratories Inc., United States). cDNA (0.2 μg) and specific primers [*Crh*: For 5′-GAAACTCAGAGCCCAAGTACGTTGAG-3′; Rev: 5′-GTTG TTCTGCGAGGTACCTCTCTCAG3′; *Crhr1*: For 5′-GTCCCT GACCAGCAATGTTT-3′; Rev 5′-CGGAGTTTGGTCATGAGG AT-3′; *Crhr2*: For 5′-AAGGTCCTAGGAGTGATCCGATT-3′; Rev 5′GGAGCCCACCAGAGAGTGCAG-3′; β –actin: For 5′-AACAT CATCCCTGCATCCAC-3′; Rev 5′-AGGAACACGGAAGGCCA TGC-3′; Brain- derived neurotrophic factor (*Bdnf*) For 5′-GGCCCAACGAAGAAAACCAT-3′; Rev 5′-AGCATCACC CGGGAAGTGT-3′; exon- III For 5′-TTGGAGGGCTCCTGC TTTCT-3′; Rev 5′-CTGGGCTCAATGAAGCAT CCAG3′; exon-IV For 5′-ACTGAAGGCGTGCGAGTATT-3′; Rev 5′-TGG TGGCCGATATGT ACTCC-3′; exon-VI For 5′-GATGAGA CCGGGTTCCCTCA-3′; Rev 5′-TTGTTGTCACGCTCCTG GTC-3′; (100 pm/each)] (Nair and Wong-Riley, 2016) in separate reaction (10 μL) containing real-time mixture (SYBR green super mix, Bio-Rad laboratories Inc.) was used to estimate expression with the following reaction cycle: 92°C initial denaturation (3 min), subsequent denaturation at 92°C (5 s), annealing [(5 s:*Crh* (63.4°C), *Crhr1* (62.0°C), *Crhr2* (63.6°C), B-actin (60.0°C), total *Bdnf* (59.0°C); exon-III (63.0°C); exon-IV (59.8°C); exon-VI (59.0°C)], and extension at 72°C (5 s) for 40 cycles and final extension at 72°C (10 min). Specific amplification was confirmed by dissociation and melting curve analysis (CFX-96 Touch Real-Time PCR detection system; CFX Manager version 2 software; Bio-Rad Laboratories Inc., United States). The data were normalized to the internal control and presented as the mean fold change.

### 2.5 Statistical analysis

GraphPad prism (ver 8.0) was used to plot the values (mean ± standard error of the mean (SEM)) as a graphical representation. The significant difference among the experimental groups (Ctrl, PMS, PMS+OXT) were tested with One–way Analysis of Variance (ANOVA), Two–way ANOVA for *Bdnf* Transcript variant followed by *post hoc* (Bonferroni test) analysis and Regression analysis (Sigma Stat version 11.0). Significant difference noted as (*p < 0.05, **p < 0.01, ***p < 0.001 and NS—indicate not significantly different.

## 3 Results

### 3.1 Transgenerational inheritance of PMS induced deficit in reference and working memory

**FIGURE 2:**
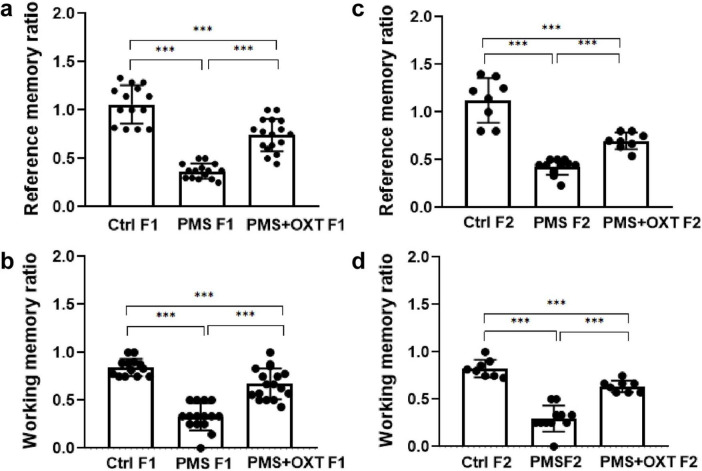
Effect of prenatal maternal stress (PMS) on F1 and F2 offspring reference and working memory. Behavioral profile in the hole board test showing that PMS reduced the reference and working memory of the F1 generation **(A,B)** of stressed offspring (PMS F1) and F2 generation **(C,D)** from the stressed maternal line (PMS F2). Exposure to oxytocin relieved the PMS-induced effect and improved the reference and working memory of F1 generation **(A,B)** of offspring (PMS+OXT F1) and F2 generation **(C,D)** from the maternal line (PMS+OXT F2). Data are represented as mean ± SE, and statistical significance is indicated by ***p < 0.001; NS, not significant.

**FIGURE 3:**
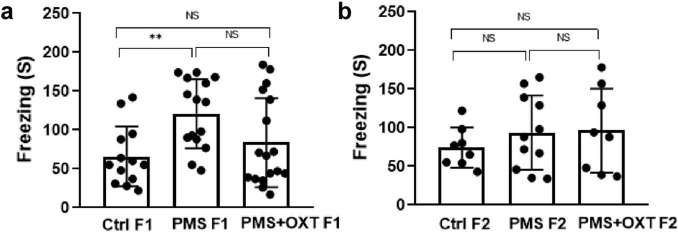
Effect of prenatal maternal stress (PMS) on anxiety-like behavior in F1 and F2 offspring Freezing behavior recorded for F1 generation **(A)** of stressed offspring (PMSF1) and F2 offspring **(B)** from the stressed maternal line (PMSF2) in the hole board arena. Exposure to oxytocin relieved PMS-induced stress in F1 offspring (PMS+OXT F1) and F2 offspring from the maternal line (PMS+OXT F2). Data are represented as mean ± SE, and statistical significance is indicated by **p < 0.01; NS, not significant.

First, we examined the PMS-induced transgenerational effect on learning and memory in F1 and F2 offspring. We found a significant difference in reference (*F*_2,44_ = 145.36; *P <* 0.001) and working memory (*F*_2,44_ = 174.82; *P <* 0.001) in the F1 offspring. *Post hoc* analysis indicated greater effects of PMS-induced deficit in reference and working memory in PMS F1 offspring. The PMSF1 offspring number of errors was higher (*P <* 0.001) than that of Ctrl and PMS+OXT; however, PMS+OXTF1 offspring made more errors than CtrlF1 offspring (*P <* 0.001) (Figures 2A,B). Further analysis showed the impact of PMS in F2 offspring, and the calculated reference (*F*_2, 26_ = 145.36; *P <* 0.001) and working memory (*F*_2,26_ = 60.625; *P <* 0.001) were significantly different among F2 offspring from Ctrl, PMS, PMS+OXT. *Post hoc* analysis indicated that PMS F2 offspring displayed more errors than Ctrl F2 and PMS+OXT F2 offspring (*P <* 0.001). In contrast, the performance of PMS+OXT F2 offspring was significantly lower than that of Ctrl F2 (Figures 2C,D). The behavioral profile of the F1 and F2 offspring in the hole board test are shown in Supplementary Figures S2, S3. In addition, anxiety-like behaviors were observed. The freezing duration of F1 offspring was significantly different (*F*_2,44_ = 4.783; *P* = 0.013). *Post hoc* comparisons indicated that PMS F1 offspring freezing duration was significantly higher than that of Ctrl F1 and PMS+OXT F1 (*P <* 0.01) offspring, and no significant difference was observed between PMS F1 and PMS+OXT F1 (*P* > 0.05) and Ctrl F1 vs. PMS+OXT F1 (*P* > 0.05) (Figure 3A). Notably, there was no significant difference among the F2 offspring (*F*_2,26_ = 0.595; *P* = 0.559), and also in all comparison between F2 offspring (*P* > 0.05) (Figure 3B). The observed behavioral data suggest that PMS induced memory deficit in F1 offspring, and was inherited to F2 offspring at some extent, which was confirmed by partial recovery by administration of oxytocin.

### 3.2 Transgenerational inheritance of H3K4me2 and H3K4me3 methylation

**FIGURE 4:**
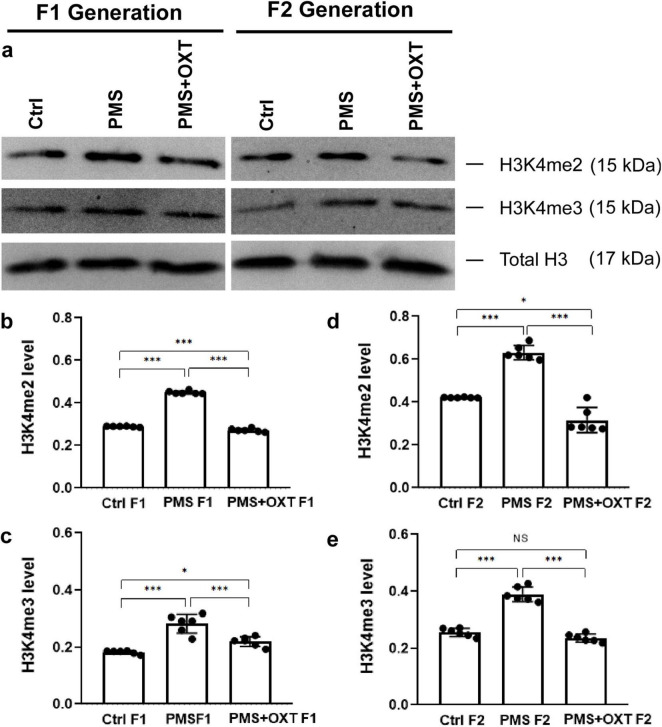
Effect of prenatal maternal stress (PMS) on H3K4me2 and H3K4me3 methylation levels in F1 and F2 offspring. Western blot analysis showed H3K4me2 and H3K4me3 methylation patterns in the F1 and F2 offspring. **(A)** PMS increases the level of methylation in F1 (PMS F1) offspring [H3K4me2 **(B)**, H3K4me3 **(C)**] and F2 (PMS F2) offspring [H3K4me2 **(D)**, H3K4me3 **(E)**]. Exposure to oxytocin reduced the PMS-induced effect and decreased the levels of H3K4me2 and H3K4me3 in F1 (PMS+OXT F1) and F2 (PMS+OXT F2) offspring. Data are represented as mean ± SE, and statistical significance is indicated by *P < 0.05; ***P < 0.001 NS – not significant.

We examined the levels of H3K4me2 and H3K4me3 to determine whether PMS induced methylation. We found that the levels of H3K4me2 [*F*_(2,17)_ = 146.259; *P* < 0.001] and H3K4me3 [*F*_(2,17)_ = 48.160, *P* < 0.001] were significantly different among F1 offspring. Further analysis showed that PMS significantly increased H3K4me2 and H3K4me3 methylation, which was significantly higher than that in the Ctrl F1 (*P* < 0.001) and PMS+OXT F1 (*P* < 0.001) offspring, and there was a significant difference between CtrlF1 and PMS+OXT F1 (*P* < 0.05) (Figures 4A–C). We observed a similar pattern of H3K4me2 [*F*_(2,17)_ = 13.023; *P* < 0.01] and H3K4me3 [*F*_(2,17)_ = 38.908, *P* < 0.001] methylation status in F2 offspring. The observed changes in the levels of H3K4me2 and H3K4me3 were significantly higher in the PMS F2 offspring than in CtrlF2 and PMS+OXT F2 offspring (*P* < 0.001). However, no significant difference was detected between CtrlF2 and PMS+OXT F2 (*P* > 0.05) in H3K4me2 or H3K4me3 levels (Figures 4A,D,E). The observed methylation status suggests that PMS induced changes possibly inherited from the F1 to F2 offspring.

### 3.3 Transgenerational inheritance of H3K4me2/me3 methylation in CRH promoter

**FIGURE 5:**
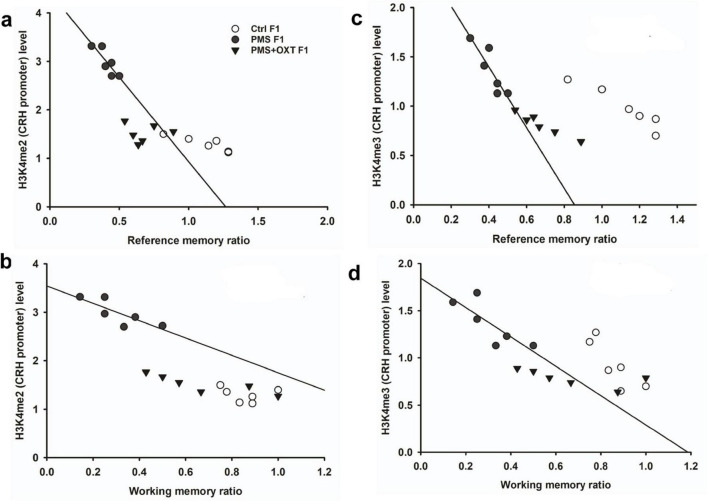
Prenatal maternal stresses (PMS) alters the levels of H3K4me2 and H3k4me3 methylation in the CRH promoter. A chromatin immunoprecipitation (ChIP) assay followed by quantitative real-time PCR analysis showed H3K4me2 and H3K4me3 methylation status in the CRH promoter. Further analysis showed that level of H3K4me2 **(A,B)** and H3K4me3 **(C,D)** significantly influenced reference memory working memory, respectively, in F1 offspring. PMS increased the level of methylation in the CRH promoter of PMSF1 and oxytocin exposure decreased methylation in PMS+OXT F1 offspring.

**FIGURE 6:**
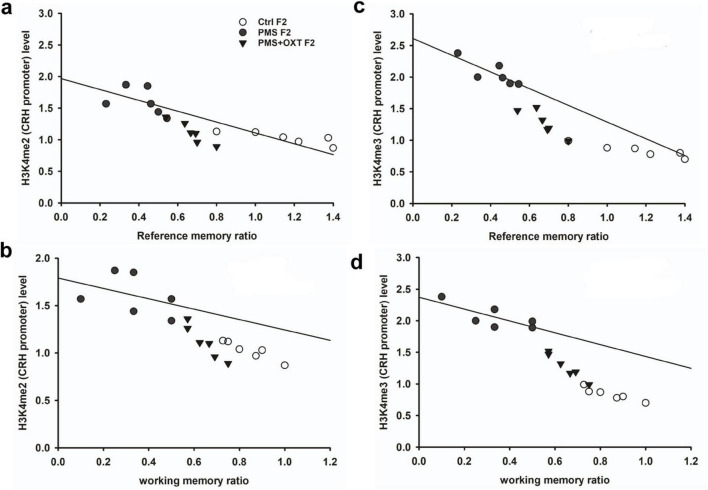
Prenatal maternal stresses (PMS) alters the levels of H3K4me2 and H3k4me3 methylation in the CRH promoter. A chromatin immunoprecipitation (ChIP) assay followed by quantitative real-time PCR analysis showed H3K4me2 and H3K4me3 methylation status in the CRH promoter. Further analysis showed that level of H3K4me2 **(A,B)** and H3K4me3 **(C,D)** significantly influenced reference memory working memory, respectively, in F2 offspring. PMS increased the level of methylation in the CRH promoter of PMSF2 offspring, and oxytocin exposure decreased methylation in PMS+OXT F2 offspring.

Subsequently, we examined whether PMS induced H3K4me2/ H3K4me3 methylation of the CRH promoter. In F1 offspring, we found that the levels of H3K4me2 [*F*_2, 17_ = 56.099; *P* < 0.001) and H3K4me3 (*F*_2, 17_ = 28.809, *P* < 0.001) were significantly different in the CRH promoter. Furthermore, PMS-induced H3K4me2 and H3K4me3 methylation in the CRH promoter was significantly higher in PMS offspring than in Ctrl F1 (*P* < 0.001) and PMS+OXT F1 (*P* < 0.001) offspring, but no significant difference was detected between CtrlF1 and PMS+OXT F1 (*P* > 0.05) (Supplementary Figures S4A,B). A similar pattern of methylation status was observed in the CRH promoter of F2 offspring. The level of H3K4me2 [*F*_(2,17)_ = 11.160; *P* < 0.001] and H3K4me3 [*F*_(2,17)_ = 38.521, *P* < 0.001] were significantly different among F2 offspring. H3K4me2 and H3K4me3 levels were significantly higher in the PMS F2 offspring than in the CtrlF2 and PMS+OXT F2 offspring (*P* < 0.001). However, no significant difference was detected between CtrlF2 and PMS+OXT F2 (*P* > 0.05) in H3K4me2 levels, but not in H3K4me3 levels (*P* < 0.05) (Supplementary Figures S4C,D). Furthermore, we have observed negative correlation in the level of methylation with reference/ working memory in F1, and F2 offspring. We found that elevated level of H3K4me2, H3K4me3 methylation significantly reduced reference (Figures 5A,C) and working memory (Figures 5B,D) in F1 offspring. Similar effect was observed in F2 offspring reference (Figures 6A,C) and working memory (Figures 6B,D). The detected methylation level demonstrates that PMS induced H3K4me2 and H3K4me3 methylation status of the CRH promoter inherited from F1 to F2 offspring.

### 3.4 Transgenerational inheritance of regulation of Crh expression

We examined the effects of CRH promoter methylation on *Crh* mRNA levels. We found a significant difference in the level of *Crh* mRNA between the F1 offspring [*F*_(2,17)_ = 321.516, *P <* 0.001]. Post-hoc analysis suggested that *Crh* mRNA levels were significantly higher in PMS F1 offspring than in CtrlF1 and PMS+OXT F1 offspring (*P <* 0.001). However, the *Crh* mRNA levels were significantly higher in the PMS+OXT F1 group than in the CtrlF1 group (*P* < 0.05) (Supplementary Figure S5A). A similar pattern was observed in F2 offspring [*F*_(2,17)_ = 175.331, *P <* 0.001]. Accordingly, the level of *Crh* mRNA was significantly higher in PMS F2 offspring than in CtrlF2 and PMS+OXT F2 offspring (*P <* 0.001), however, the level was significantly higher in PMS+OXT F2 offspring than in Ctrl F2 offspring (*P <* 0.001) (Supplementary Figure S5D). The observed data demonstrate that exposure to oxytocin may counteract PMS induced transcriptional activation of *Crh.*

*Crh* facilitates regulation possibly through *Crhr1* and *Crhr2*; therefore, we examined the levels of *Crh* receptors. The estimated level of *Crhr1* in F1 offspring was significantly different between the F1 offspring [*F*_(2,17)_ = 30.249, *P <* 0.001]. *Post hoc* analysis showed that PMS influenced the expression of *Crhr1*, which was significantly higher in PMS than in CtrlF1, PMS+OXT F1 (*P <* 0.001); however, the level of *Crhr1* in PMS+OXT was not different from that in Ctrl F1 (*P* > 0.05) (Supplementary Figure S5B). The effect of PMS was observed in the F2 offspring; thus, we observed a significant difference between the experimental groups [*F*_(2,17)_ = 32.920, *P* < 0.001]. The estimated levels in PMS F2 offspring were significantly higher than those in Ctrl F2 and PMS+OXT F2 (*P* < 0.001), but there was no significant difference between the Ctrl F2 and PMS+OXT F2 offspring (*P* > 0.05) (Supplementary Figure S5E).

**FIGURE 7:**
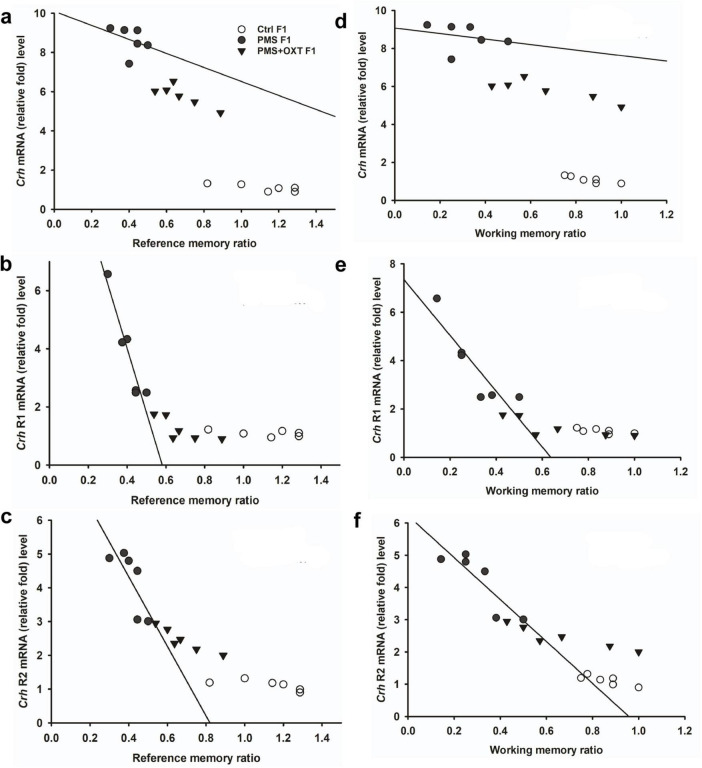
Prenatal maternal stress (PMS) alters the expression of corticotrophin-releasing hormone (*Crh* mRNA) and its receptors (*Crhr1* and *Crhr2*). Quantitative real– time PCR analysis showing the expression pattern of *Crh, Crhr1 Crhr2* in F1 offspring. The analysis showed that PMS significantly increased the levels of *Crh, Crhr1 Crhr2* and decreased the reference **(A–C)** and working memory **(D–F)** in PMSF1 offspring, and oxytocin exposure decreased the levels and increased memory in PMS+OXT F1 offspring.

**FIGURE 8:**
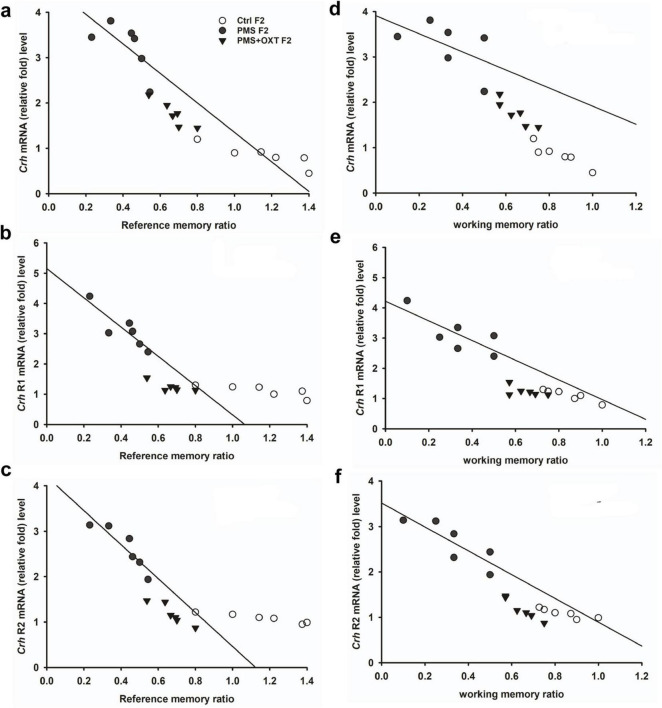
Prenatal maternal stress (PMS) alters the expression of corticotrophin-releasing hormone (*Crh* mRNA) and its receptors (*Crhr1* and *Crhr2*). Quantitative real–time PCR analysis showing the expression pattern of *Crh, Crhr1 Crhr2* in F2 offspring. The analysis showed that PMS significantly increased the levels of *Crh, Crhr1 Crhr2* expression, and decreased the reference **(A–C)**, working memory **(D–F)** in PMSF2 offspring, and oxytocin exposure decreased the levels and increased memory in PMS+OXT F2 offspring.

Subsequently, we found that the expression of *Crhr2* was significantly different among the experimental groups in F1 offspring [*F*_(2,17)_ = 46.008, *P* < 0.001]. PMS significantly induced the expression of *Crhr2*; the estimated level in the PMS F1 offspring was higher than that in Ctrl F1 and PMS+OXT F1 offspring (*P* < 0.001), but the level was significantly higher in the PMS+OXT F1 offspring (P < 0.01) (Supplementary Figure S5C). A similar pattern of *Crhr2* expression in F2 offspring was observed in F2 offspring [*F*_(2,17)_ = 16.736, *P* < 0.001]. Expression in PMS F2 offspring was higher than in Ctrl F2 and PMS+OXT F2 offspring (*P* < 0.001), whereas a higher level of *Crhr2* was observed in PMSF2 offspring than in Ctrl F2 offspring (*P* < 0.001) (Supplementary Figure S5F). Interestingly, we found that elevated level of *Crh, Crh R1* and *Crh R2* expression significantly reduced the reference and working memory in F1 and F2 generation. We have observed a strong significant negative association between reference memory [*Crh* (*r* = -0.71, *P* < 0.001), *Crh R1* (*r* = -0.23, *P* > 0.05), *Crh R2* (*r* = -0.417, *P* < 0.01)] and working memory [*Crh* (*r* = -0.23, *P* > 0.05), *Crh R1* (*r* = -0.42, *P* < 0.05), *Crh R2* (*r* = -0.817, *P* < 0.001)] in F1 offspring (Figure 7). Similar pattern of relationship was observed in F2 offspring’s reference memory [*Crh* (*r* = -0.89, *P* < 0.001), *Crh R1* (*r* = -0.67, *P* < 0.001), *Crh R2* (*r* = -0.84, *P* < 0.01)] and working memory [*Crh* (*r* = -0.78, *P* < 0.001), *Crh R1* (*r* = -0.86, *P* < 0.001), *Crh R2* (*r* = -0.88, *P* < 0.001)] (Figure 8).

### 3.5 Transgenerational inheritance of regulation of corticosterone

**FIGURE 9:**
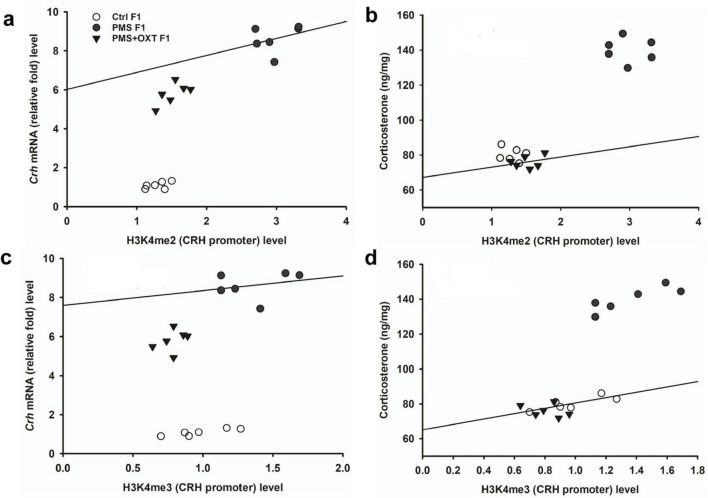
Effect of prenatal maternal stress (PMS)-associated H3K4me2, H3 K4me3 methylation CRH promoter significantly changes *Crh* mRNA and corticosterone (CORT) level in F1 generations. The level of H3K4me2, H3 K4me3 methylation significantly influences the level of *Crh* mRNA **(A,C)** and CORT **(B,D)** in F1 generation was significantly increased by PMS F1 offspring, but exposure to oxytocin attenuated the PMS induced effect and reduced the *Crh* mRNA, CORT level in PMS+OXT F1 offspring.

**FIGURE 10:**
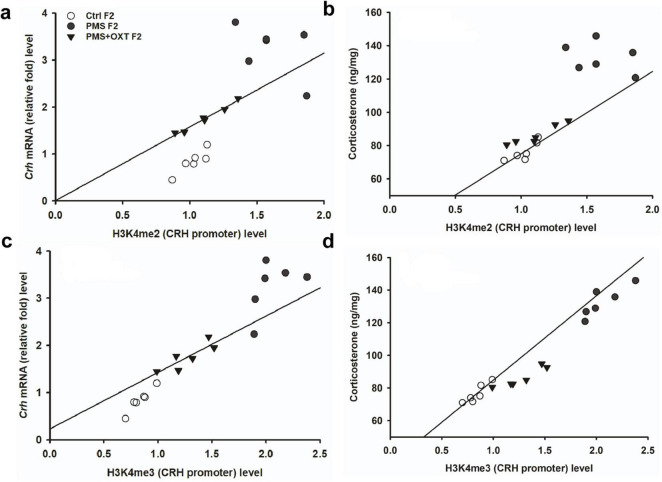
Effect of prenatal maternal stress (PMS)-associated H3K4me2, H3 K4me3 methylation CRH promoter significantly changes *Crh* mRNA and corticosterone (CORT) level in F2 generations. The level of H3K4me2, H3 K4me3 methylation significantly influences the level of *Crh* mRNA **(A,C)** and CORT **(B,D)** in F2 generation was significantly increased by PMS F2 offspring, but exposure to oxytocin attenuated the PMS induced effect and reduced the *Crh* mRNA, CORT level in PMS+OXT F2 offspring.

To investigate the effect of PMS induction on stress hormone levels, we estimated CORT levels in the amygdala. We found that PMS significantly increased CORT level in F1 and F2 offspring. The level of CORT was significantly altered in F1 [*F*_(2,17)_ = 303.106, *P* < 0.001] and F2 [*F*_(2,17)_ = 183.7, *P* < 0.001] offspring. In comparison, the level of CORT was significantly higher in PMS F1 and PMS F2 offspring (*P* < 0.001) than in Crtl F1, CrtlF2, PMS+OXT F1, and PMS+OXT F2 offspring, but significant differences were not detected between CrtlF1, CrtlF2, PMS+OXT F1, and PMS+OXTF2 (*P* > 0.05) offspring (Supplementary Figure S6). Further analysis revealed that level of methylation of H3K4me2/ H3K4me3 associated with level of *Crh* mRNA and CORT. The regression analysis showed significant positive association between H3K4me2/ H3K4me3 methylation with *Crh* mRNA (*r* = 0.67, *P* < 0.001; *r* = 0.53, *P* < 0.01) and CORT (*r* = 0.52, *P* < 0.01; *r* = 0.51, *P* < 0.01) (Figure 9) in F1 offspring. Similarly, significant positive association detected between H3K4me2/ H3K4me3 methylation with *Crh* mRNA (*r* = 0.86, *P* < 0.001; *r* = 0.85, *P* < 0.001) and CORT (*r* = 0.83, *P* < 0.001; *r* = 0.89, *P* < 0.001) in F2 offspring (Figure 10).

### 3.6 Transgenerational inheritance of regulation of Bdnf splices variant expression

**FIGURE 11:**
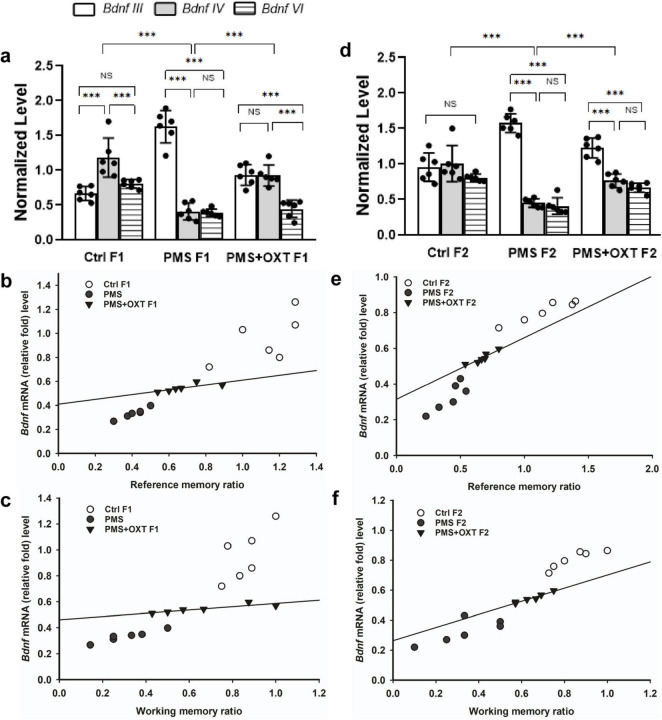
Effect of prenatal maternal stress (PMS) regulation of *Bdnf* splice variant and total *Bdnf* expression in the F1 and F2 generations. Quantitative real–time PCR analysis showed that PMS/oxytocin mediated alterations in the expression of *Bdnf* exon—III, IV and exon—VI transcripts. The analysis showed that PMS differentially altered the expression, level of exon-III increased, and exon-IV and VI decreased in **(A)** F1 (PMS) and **(D)** F2 (PMS) offspring. Administration of oxytocin minimized the PMS-induced effect, decreased exon-III, and increased exon-IV levels in **(A)** F1 (PMS+OXT) and **(D)** F2 (PMS+OXT) offspring. Furthermore, the level of total *Bdnf* was decreased by PMS in the PMS F1 and PMS F2 offspring, and oxytocin administration reverse the PMS induced effect. Correlation analysis showed that the level of total *Bdnf* mRNA significantly associated reference **(B,E)**, working memory **(C,F)** in F1 and F2 offspring. Data are represented as mean ± SE, and statistical significance is indicated by **p < 0.01; ***p < 0.001; NS, not significant.

We examined the influence of CRH on BDNF by examining the levels of the *Bdnf* variants. We detected contrasting patterns in the mRNA levels of exon-III and exon-IV. A two-way ANOVA revealed that there was a significant difference between the experimental groups with exon III, IV and VI [*F*_(2,53)_ = 4.024, *P* < 0.05], within the group between exon-III IV and VI [*F*_(2,53)_ = 103.08, *P* < 0.010], and there was a significant interaction between group and exon-III/IV/VI [*F*_(2,53)_ = 31.77, *P* < 0.01]. *Post hoc* analysis showed that PMS significantly (*P* < 0.001) up-regulated the expression of exon-III and down regulated exon-IV and VI in PMS F1 (*P* < 0.001), but no significant difference was observed in the levels of exon-IV and VI (*P* > 0.05). In contrast, the level of exon-IV was restored in PMS+OXT F1. Thus, the level of exon-III, IV were significantly higher than those of exon-VI (*P <* 0.001), but there was no significant difference between exon-III and IV (*P* > 0.05). Note to mention that OXT treatment significantly restore the level of exon-IV, which was higher than that of PMS F1 (*P* < 0.001) and Ctrl F1(*P* < 0.001) (Figure 11A). Differential expression of splice variants significantly alter the level of *Bdnf* total mRNA [*F*_(2,17)_ = 41.21, *P* < 0.001]. PMS significantly reduced the level (*P* < 0.001) compared to than CtrlF1 and PMS+OXT F1, however, there was significant difference between Ctrl F1 and PMS+OXT F1 (*P* < 0.001), and PMS F1 and PMS+OXT F1 (*P* < 0.01). Further analysis showed that *Bdnf* mRNA level significantly influenced the behavior, we found significant positive correlation between *Bdnf* mRNA and reference memory (*r* = 0.48, *P* < 0.05), and working memory (*r* = 0.43, *P* < 0.05) in F1 generation (Figures 11B,C).

A similar pattern was observed in F2 offspring, and a significant difference was detected between experimental groups with exon III, IV and exon-VI [*F*_(2,53)_ = 4.45, *P* < 0.05]. Significant differences were observed between exon-III, IV and VI within the group [*F*_(2,53)_ = 49.99, *P* < 0.001], and there was a significant interaction between group and exon-III/ IV/VI [*F*_(2,53)_ = 52.81, *P* < 0.001]. Furthermore, we found that the level of exon-III was increased and that of exon-IV, and VI was decreased in PMS F2 offspring, but no significant difference was detected between exon-IV and VI in CtrlF2 or PMS F2, PMS+OXT F2 offspring (Figure 11D). However, a significant difference in total *Bdnf* was observed [*F*_(2,17)_ = 88.56, *P* < 0.001]. We found that level of total *Bdnf* was significantly reduced PMS F2 (*P* < 0.001) compared to CtrlF2 and PMS+OXT F2, and there was significant difference between Ctrl F2 and PMS+OXT F2 (*P* < 0.001), and PMS F2 and PMS+OXT F2 (*P* < 0.001). Correlation analysis demonstrated a significant positive association between *Bdnf* level to reference memory (*r* = 0.638, *p* = 0.025) and working memory (*r* = 0.757, *p* = 0.004) in F2 offspring (Figures 11E,F).

**FIGURE 12:**
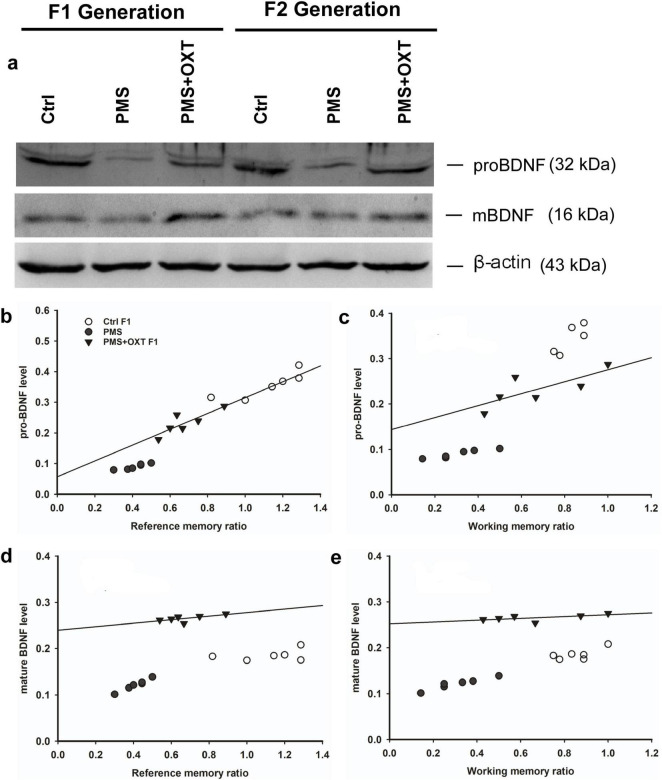
Effect of prenatal maternal stress (PMS) alter the level of pro-BDNF and mature BDNF in the F1 and F2 generations. **(A)** Representative western blots showing the expression levels of pro-and mature BDNF in the F1 and F2. The analysis showed that PMS significantly reduced the level of pro-and mature BDNF in F1 offspring, which is positively correlated with reference **(B,D)**, working memory **(C,E)** in F1 offspring. Administration of oxytocin minimized the PMS-induced effect, restored the level of pro-and mature BDNF in F1 (PMS+OXT) and F2 (PMS+OXT) offspring.

**FIGURE 13:**
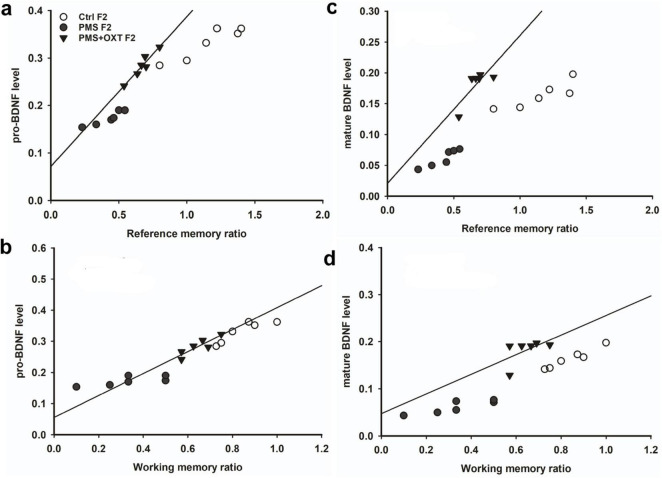
Effect of prenatal maternal stress (PMS) alter the level of pro-BDNF and mature BDNF in the F1 and F2 generations. **(A)** The analysis showed that PMS significantly reduced the level of pro-and mature BDNF in F2 offspring, which is positively correlated with reference **(B,D)**, working memory **(C,E)** in F2 offspring. Administration of oxytocin minimized the PMS-induced effect, restored the level of pro-and mature BDNF in F1 (PMS+OXT) and F2 (PMS+OXT) offspring.

Further, we examined whether differential levels of exon-III, IV and exon-VI- mRNA influence the translation of BDNF protein. Significant differences were found between the experimental groups in the F1 [*F*_(2,17)_ = 127.144, *P* < 0.001] and F2 [*F*_(2,17)_ = 42.556, *P* < 0.001] offspring. PMS significantly influenced pro-BDNF levels in PMS F1 and PMS F2 offspring, which were lower than those in CtrlF1, CtrlF2, PMS+OXT F1, and PMS+OXTF2 (*P* < 0.001) offspring; however, the difference between CtrlF1 vs. PMS+OXT F1 and Ctrl2 vs. PMS+OXT F2 was not significant (*P* > 0.05) (Supplementary Figures S7B,C). In addition, we found positive correlation between pro-BDNF with reference and working memory. The analysis showed that elevated level of pro-BDNF positively associated with reference memory (F1: *r* = 0.742, *P* < 0.001; F2: *r* = 0.88, *P* < 0.001), working memory (F1: *r* = 0.674, *P <* 0.001; F2: *r* = 0.84, *P* < 0.001) in F1 and F2 generation (Figures 12A–C, 13A,B). Similar impact was observed in mature BDNF, significant difference was observed between the experimental groups in F1 [*F*_(2,17)_ = 252.53, *P* < 0.001] and F2 [*F*_(2,17)_ = 60.57, *P* < 0.001]. Level of mature BDNF in PMS F1 and PMS F2 offspring was significantly lower than CtrlF1, CtrlF2, PMS+OXT F1, and PMS+OXTF2 (*P* < 0.001) offspring, and there was a difference between Ctrl F1 vs. PMS+OXT F1 (*P* < 0.001), but not in F2 offspring Ctrl F2 vs. PMS+OXT F2 was not significant (*P* > 0.05) (Supplementary Figures S7D,E). Similar to pro-BDNF, level of mature BDNF positively associated with reference memory (F1: *r* = 0.312, *P* < 0.05; F2: *r* = 0.86, *P* < 0.001), working memory (F1: *r* = 0.28, *P >* 0.05; F2: *r* = 0.81, *P* < 0.001) in F1 and F2 generation (Figures 12D,E, 13C,D). The results suggest that PMS influences the expression of exon-III exon-IV, exon-VI variations, which alter the level of pro-and mature BDNF protein. Observed reference, working memory could be correlated with the level of pro-and mature BDNF.

## 4 Discussion

Recent studies have shown that many environmental, social and metabolic stressors affect the next generation(s) physiology and behavior. The long-term effects of maternal stress are not well established, particularly in offspring (McDonald et al., 2023). Therefore, the present study aimed to examine maternal line-mediated transgenerational epigenetic inheritance in an animal model of prenatal maternal stress. Indeed, we found that reference and working memory were reduced in PMSF1 and PMSF2 offspring, which may be associated with the effect of maternal stress (F0) on F1 offspring (Grundwald and Brunton, 2015; McDonald et al., 2023). Oxytocin treatment rescued reference and working memory deficit in PMS+OXT F1 and PMS+OXT F2 offspring. Oxytocin exposure possibly facilitate the interaction between oxytocin and CORT possibly regulate the stress response (Matsushita et al., 2019; Young et al., 2021), inhibition of maternal CORT response to stressors possibly attenuates or reduces several maternal stress induced effects in offspring (Ma et al., 2020; Thirtamara Rajamani et al., 2024), thus, the oxytocin exposed offspring showed improved memory. Furthermore, the observed memory deficits in the PMS F1/PMS F2 offspring could be linked to freezing. Our observations are consistent with earlier reports that documented that exposure to oxytocin exerts antidepressant and anxiolytic effects (Naja and Aoun, 2017; Yoon and Kim, 2022). Early life stress has been known to induce functional and developmental changes in the amygdala, i.e., dynamic changes in morphology, volume, cell proliferation and other physiology (Kraszpulski et al., 2006; Fareri and Tottenham, 2016). Extensive studies in the amygdala suggest that the activation of oxytocin exerts inhibitory effects within the amygdala through GABAergic interneuron to decrease anxiety, stress and facilitate social behavior (Labuschage et al., 2010). Furthermore, activation of the oxytocin receptor specifically in amygdala facilitate a range of social behavior (Fam et al., 2018, 2024) and regulates both bottom-up and top-down emotion (Xin et al., 2020). Therefore, we examined PMS/oxytocin treatment mediated epigenetic changes in the amygdala in this study. The induction of CORT by PMS may be a critical factor in the observed freezing and memory deficit (Conrad et al., 2024). We found that CORT levels were higher in PMS F1, PMS F2, than CtrlF1, CtrlF2, PMS+OXT F1, and PMS+OXT F2. The amygdala is a key integrating region that connects the emotional, endocrine and autonomic responses to stress. In addition, daily circulating levels of CORT in the amygdala in response to stress induce anxiety-like behavior and delivery of corticosterone to the amygdala after stress prevents stress induced effects (Venkova et al., 2010; Chakraborty et al., 2020). Thus, the observed higher level of CORT in PMS F1 offspring could be associated with *in utero* transmission of stress from the mother (F0) (Erisman et al., 1990; Bingham et al., 2013) and then from PMS F1 to PMS F2 offspring. The interplay between OXT and CRH reveals that OXT treatment elicits stress resilience and may minimize long-term effects in individuals (Li et al., 2019; Zhang et al., 2023). Therefore, the CORT level was reduced in the PMS+OXT F1 and PMS+OXT F2 groups, which showed less freezing and improved memory. Maternal stress is known to alter developmental programs, including epigenetic memory, and generate transgenerational stress lineages and behavioral phenotypes (Darnaudéry and Maccari, 2008; Ambeskovic et al., 2019). Epigenetic modification is a critical molecular mechanism that regulates developmental, cellular, and biological functions by tightly controlling gene expression (Moisiadis et al., 2017). At this point, global H3K4me2 and H3K4me3 methylation status is sensitive to social and environmental stressors (Lindeman et al., 2010). Specifically, maternal stress has been proposed to alter the epigenome, and induced changes can be transmitted across generations (Dion et al., 2022). Similarly, we found that PMS increased the levels of H3K4me2 and H3K4me3 in PMS F1 and PMS F2 offspring. Our observations provide additional support to recent clinical (Cardenas et al., 2019; Wiley et al., 2023; Dieckmann and Czamara, 2024), animal studies (Babenko et al., 2015; Grundwald and Brunton, 2015; Legoff et al., 2019), and suggest that observed memory deficit could be linked with PMS-induced methylation in F1 and transgenerational inheritance in F2 offspring. Oxytocin exposure reduced the levels of H3K4me2 and H3K4me3 in PMS+OXT F1 and PMS+OXT F2 offspring, and augmented oxytocin treatment to relieve the PMS-induced effect (Ma et al., 2020; Matsushita et al., 2019; Janeček and Dabrowska, 2019) and improve memory (Flanagan et al., 2018; Thirtamara Rajamani et al., 2024; Walia et al., 2024).

Oxytocin is known to regulate corticotropin-releasing hormone (CRH) and orchestrate feedback mechanisms of the stress response, neuronal circuitry, and synaptic plasticity (Janeček and Dabrowska, 2019). Stress-induced methylation of the CRH promoter is considered a key factor in the regulation of the HPA axis and is associated with transcriptional regulation of CORT (Zhou and Fang, 2018). We observed increased levels of H3K4me2 and H3K4me3 in the CRH promoter of PMS F1 and PMS F2 offspring compared to CtrlF1, CtrlF2/ PMS+OXT F1, and PMS+OXT F2 offspring. Elliott et al. (2010) reported that higher levels of CRH mRNA significantly associated with decreased promoter CpG methylation. However, recent studies have documented that DNA methylation not always silence the expression of genes, depending on the exact methylation site in the genome and the associated downstream transcriptional regulations (Zhu et al., 2016; Chatzittofis et al., 2021). In this study, we observed that PMS increased H3K4me2, H3K4me3 and the expression of *Crh* mRNA. The observed methylation pattern and *Crh* mRNA level possibly by two different mechanism reported earlier, possibly hypermethylation in the CRH promoter at CpGs outside the cyclicAMP response element (CRE) site (Sterrenburg et al., 2011; Chen et al., 2012) or an undetectable site for methyl-CpG-binding protein (MeCP2) at the CRH promoter lead to increased *Crh* mRNA level (McGill et al., 2006; Bhave and Uht, 2017). PMS increases the level of H3K4me2 and H3K4me3 methylation in the CRH promoter through intergenerational inheritance in offspring PMS F1 and transgenerational inheritance in their PMS F2 offspring; thus, they display deficit in reference and working memory (Blaze and Roth, 2015; Fransquet et al., 2022; Alyamani et al., 2022). Furthermore, oxytocin treatment suppresses the release of CORT and activate the stress response system (Smith and Wang, 2014; Stevenson et al., 2023), which in turn reduces the methylation level in PMS+OXT F1 offspring and is transgenerationally inherited by PMS+OXT F2 offspring. Stressors are rapidly activate CRH neurons to release CRH as a stress response and regulate the release of CORT, which in turn regulates CRH through a feedback mechanism (Kim et al., 2019) involving H3K4me2 and H3K4me3. We found a significant correlation between the levels of H3K4me2 and H3K4me3 in the CRH promoter and the level of *Crh* mRNA in F1 and F2 offspring, which could be linked to their behavioral phenotype (Zhou and Fang, 2018; Kassotaki et al., 2021; Alyamani et al., 2022). Previous studies have documented that *Crh* predominantly acts through *Crh* receptors (*Crhr1/ Crhr2*) to produce anxiety/depression-like behaviors (Bakshi et al., 2002; Paretkar and Dimitrov, 2018) and its antagonist resilience stress (Holsboer and Ising, 2008; Menke, 2024). The roles of *Crhr1* and *Crhr2* in regulating anxiety/anxiolytic behavior are unclear; *Crhr1* mediates anxiety—like behavior (Müller et al., 2003; Wang et al., 2012), and its knockdown reduces anxiety—like behavior (Smith et al., 1998).

In line with this, we found that the levels of *Crhr1* and *Crhr2* mRNA were higher in PMS F1 and PMS F2 offspring, and activated Crhr1 and *Crhr2* in PMS F1; PMS F2 offspring might exert stress and reduce reference and working memory (Greetfeld et al., 2009; Pournajafi-Nazarloo et al., 2009). CRH regulates positive/negative feedback mechanisms through its receptors and mediates the stress response/adaptive plasticity, in which BDNF is also involved (Hu et al., 2020; Kussainova et al., 2022). Notably, stress can induce long-lasting alterations through different programs, including the induction of splice variants (St-Cyr and McGowan, 2015), and alter *Bdnf* splice variants, synaptic plasticity, and behavior. Similar to other animal models (Chen et al., 2019; Begni et al., 2020; Kamaladevi and Rajan, 2023), PMS differentially alters *Bdnf* variants. HPA axis activation is known to regulate the transcription of *Bdnf* (Yuluğ et al., 2009), level of stress and CORT can selectively regulate the transcription of *Bdnf* exons (Murgatroyd et al., 2009; Hansson et al., 2006). The level of *Bdnf* exon-III mRNA was increased, but exon-IV and VI mRNA were reduced in PMS F1 and PMS F2 offspring compared to those in CtrlF1, CtrlF2/ PMS+OXT F1, and PMS+OXT F2 offspring Exon-III/IV variation is known to critically control the *Bdnf* total transcript and subsequently the level of protein (Tsankova et al., 2006; Nair et al., 2007). In contrast, exon-VI transcripts are transported to the distal dendritic compartments and released in response to stress/stimuli to regulate neuronal activity, while exon transcripts III and IV are localized in the soma and proximal region (Chiaruttini et al., 2009). A recent study demonstrate that stress induced epigenetic changes in the BDNF promoter reduced the transcripts IV and VI. This has been recognized as a key transcript in maintaining *Bdnf* levels and secretion at synapses, which may be correlated with plasticity and behavior (Mallei et al., 2015). In line with this, the levels of both pro-BDNF and mature BDNF were significantly lower in the PMS F1 and PMS F2 offspring than in the CtrlF1, CtrlF2/ PMS+OXT F1 and PMS+OXT F2 offspring. BDNF regulates a wide range of functions. Previous studies have documented that the intracellular conversion of pro-*BDNF* to mature *BDNF* is controversial and differs with developmental time points (Matsumoto et al., 2008; Yang et al., 2014). However, upon secretion into the extracellular matrix to mediate their effects, pro-and mature *BDNF* interact with their cognate receptors tropomyosin, related kinase receptor B (TrkB) and p75 neurotrophin receptor (p75NTR), respectively (Thomas and Davies, 2005). Their interaction effectively regulate long-term potentiation (LTP) (Pang et al., 2016), inhibits of GABAergic transmission (Riffault et al., 2014) and supports synaptic plasticity, learning and memory (Sakata et al., 2010; Orefice et al., 2013; Zheng et al., 2016). Thus, the observed changes in BDNF levels in the F1 and F2 offspring could be linked to their behavioral phenotypes (Lu et al., 2008; Jaehne et al., 2023; Persaud and Cates, 2023).

## 5 Conclusion

The present study summarizes that PMS induces heritable changes in global H3K4me2 and H3K4me3 methylation, which affect the level of methylation in the CRH promoter. Upregulation of *Crh* and its receptor was accompanied by methylation of the CRH promoter, whereas upregulation of CRH caused a decrease in BDNF protein expression by differentially regulating *Bdnf* exon-III, IV and VI transcripts. Furthermore, oxytocin exposure inhibits PMS induced effects that regulate synaptic plasticity, learning, and memory. Our data suggest that F0 stress alters the methylation status of H3K4me2 and H3K4me3 in F1 offspring, and altered epigenetic changes are transgenerationally inherited from F1 to F2 offspring through the maternal line. We did not examine maternal (F0) stress response or behavior, and did not compare it with male offspring, which is a limitation of our study.

## Data Availability

The original contributions presented in the study are included in the article/Supplementary material, further inquiries can be directed to the corresponding author.
