# Supplementary material for: Prenatal maternal life adversity impacts on learning and memory in offspring: implication to transgenerational epigenetic inheritance
Source: Front Neurosci. 2025 Feb 13;19:1518046. doi: 10.3389/fnins.2025.1518046 (PMC11865043; doi:10.3389/fnins.2025.1518046)
Supplement: Supplementary file 1 [file Data_Sheet_1.pdf]

Supplementary Material

Prenatal maternal life adversity impacts on learning and memory in offspring: Implication to transgenerational epigenetic inheritance

Prince David Adeline Dorothy and Koilmani Emmanuvel Rajan*

Behavioural Neuroscience Laboratory, Department of Animal Science
Bharathidasan University, Tiruchirappalli, India.

*Correspondance: KE Rajan (emmanuvel1972@yahoo.com)

Method

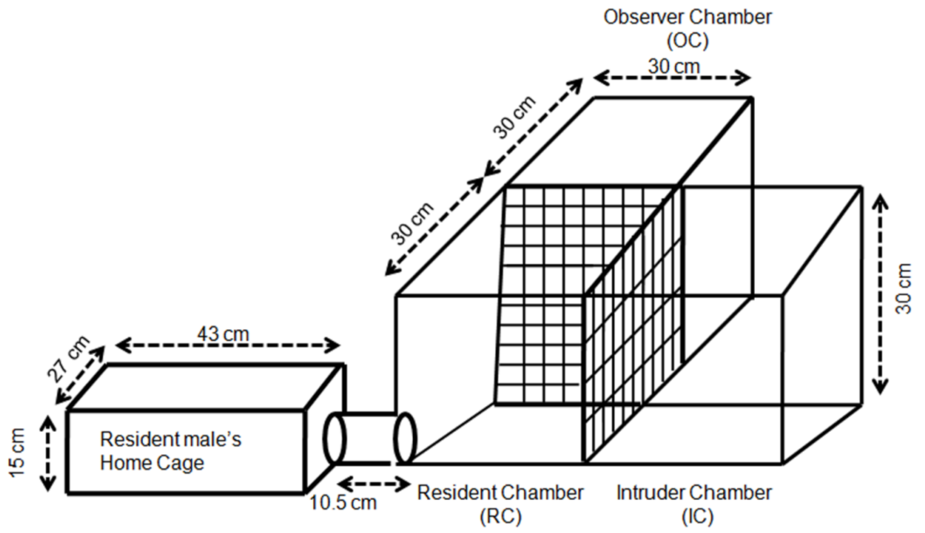

Supplementary Figure 1. Diagram showing the apparatus to induce gestational stress through social defeat observation (Sivasangari and Rajan, 2020)

Results

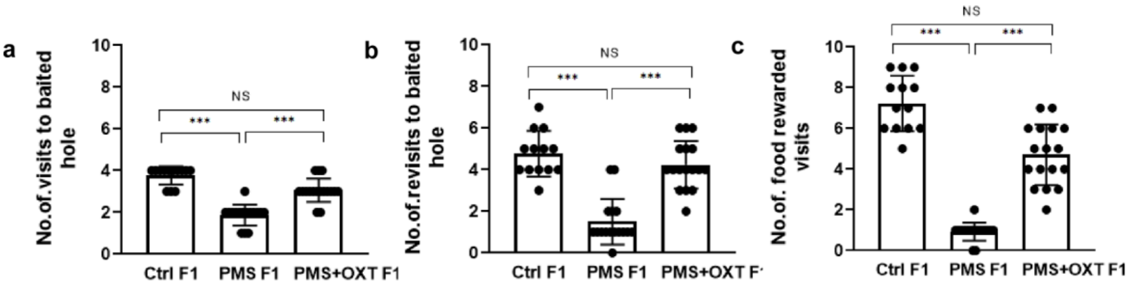

Supplementary Figure 2 Behavioural profile of F1 offspring in hole board test. Prenatal maternal stress (PMS) significantly reduced number of visits to baited hole (a), number of revisits to baited hole (b) and number of food rewarded visits (c). Exposure to oxytocin minimized the PMS-induced effect and improved their behaviour in hole board. Data are represented as mean ± SE, and statistical significance is indicated by ***p<0.001; NS, not significant.

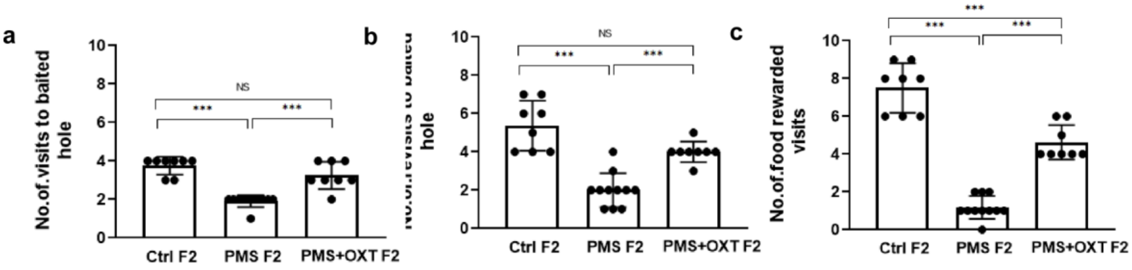

Supplementary Figure 3 Behavioural profile of F1 offspring in hole board test. Prenatal maternal stress (PMS) significantly reduced number of visits to baited hole (a), number of revisits to baited hole (b) and number of food rewarded visits (c). Exposure to oxytocin minimized the PMS-induced effect and improved their behaviour in hole board. Data are represented as mean ± SE, and statistical significance is indicated by ***p<0.001; NS, not significant.

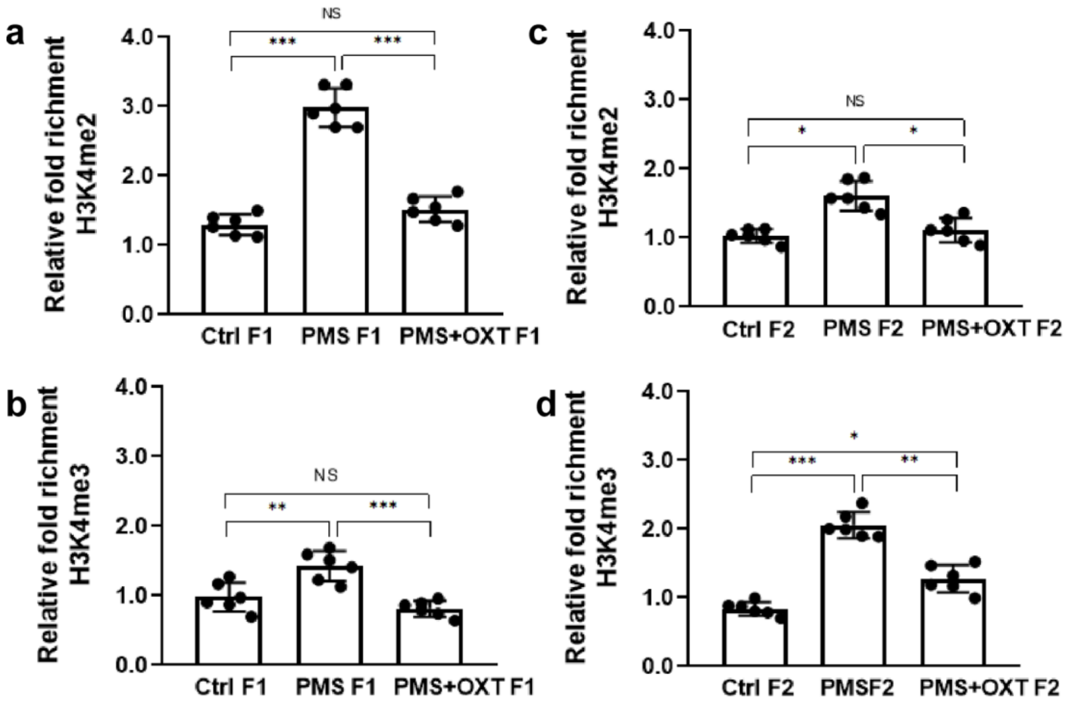

Supplementary Figure 4 Prenatal maternal stresses (PMS) alters the levels of H3K4me2 and H3k4me3 methylation in the CRH promoter. A chromatin immunoprecipitation (ChIP) assay followed by quantitative real-time PCR analysis showed H3K4me2 and H3K4me3 methylation status in the CRH promoter. The analysis showed that level of H3K4me2 **(a),** H3K4me3 (**b)** F1 offspring and H3K4me2 **(c)** H3K4me3 (d) min F2 offspring CRH promoter. PMS increased the level of methylation in the CRH promoter of PMSF1 and PMSF2 offspring, and oxytocin exposure decreased methylation in PMS+OXT F1 and PMS+OXT F2 offspring. Data are represented as mean ± SE, and statistical significance is indicated by ***$p<0.001$; NS, not significant.

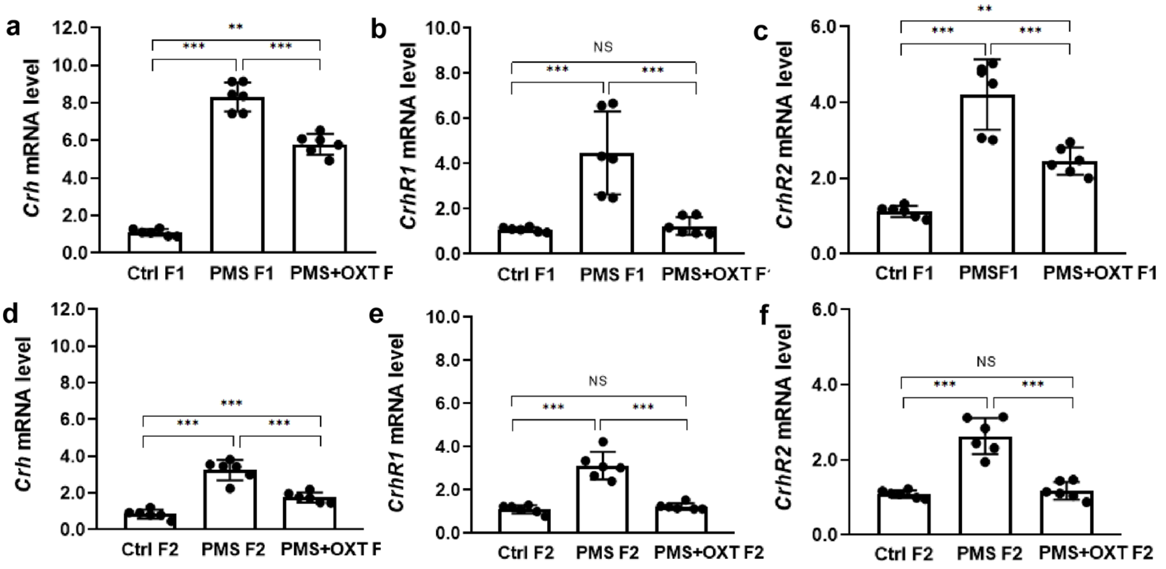

Supplementary Figure 5 Prenatal maternal stress (PMS) alters the expression of corticotrophin-releasing hormone (*Crh* mRNA) and its receptors (*Crhr1* and *Crhr2*). Quantitative real– time PCR analysis showing the expression pattern of *Crh, Crhr1 Crhr2* in F1 offspring (a,b,c) and F2 offspring (d,e,f), respectively. The analysis showed that PMS significantly increased the levels of PMSF1 and PMSF2 offspring, and oxytocin exposure decreased the levels in PMS+OXT F1 and PMS+OXT F2 offspring. Data represented as mean ± SE, statistical significance is indicated by **p<0.01 ***p<0.001; NS – Not significant.

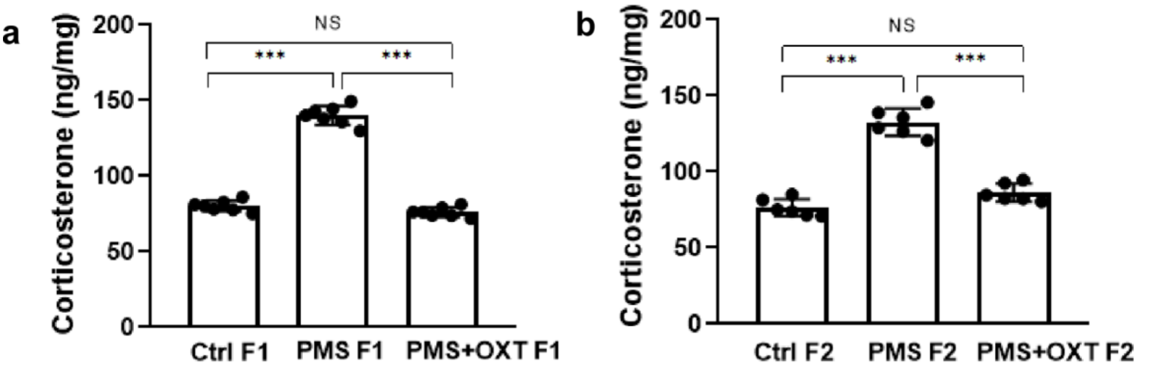

Supplementary Figure 6 Effect of prenatal maternal stress (PMS)-associated changes in corticosterone (CORT) in F1 and F2 generations. The level of CORT in F1 **(a)** and F2 **(c)** generation was significantly increased by PMS in PMS F1 and PMS F2 offspring, but exposure to oxytocin attenuated the PMS induced effect and reduced the CORT level in PMS+OXT F1 and PMS+OXT F2 offspring. Data are represented as mean ± SE, and statistical significance is indicated by ***p<0.001; NS, not significant.

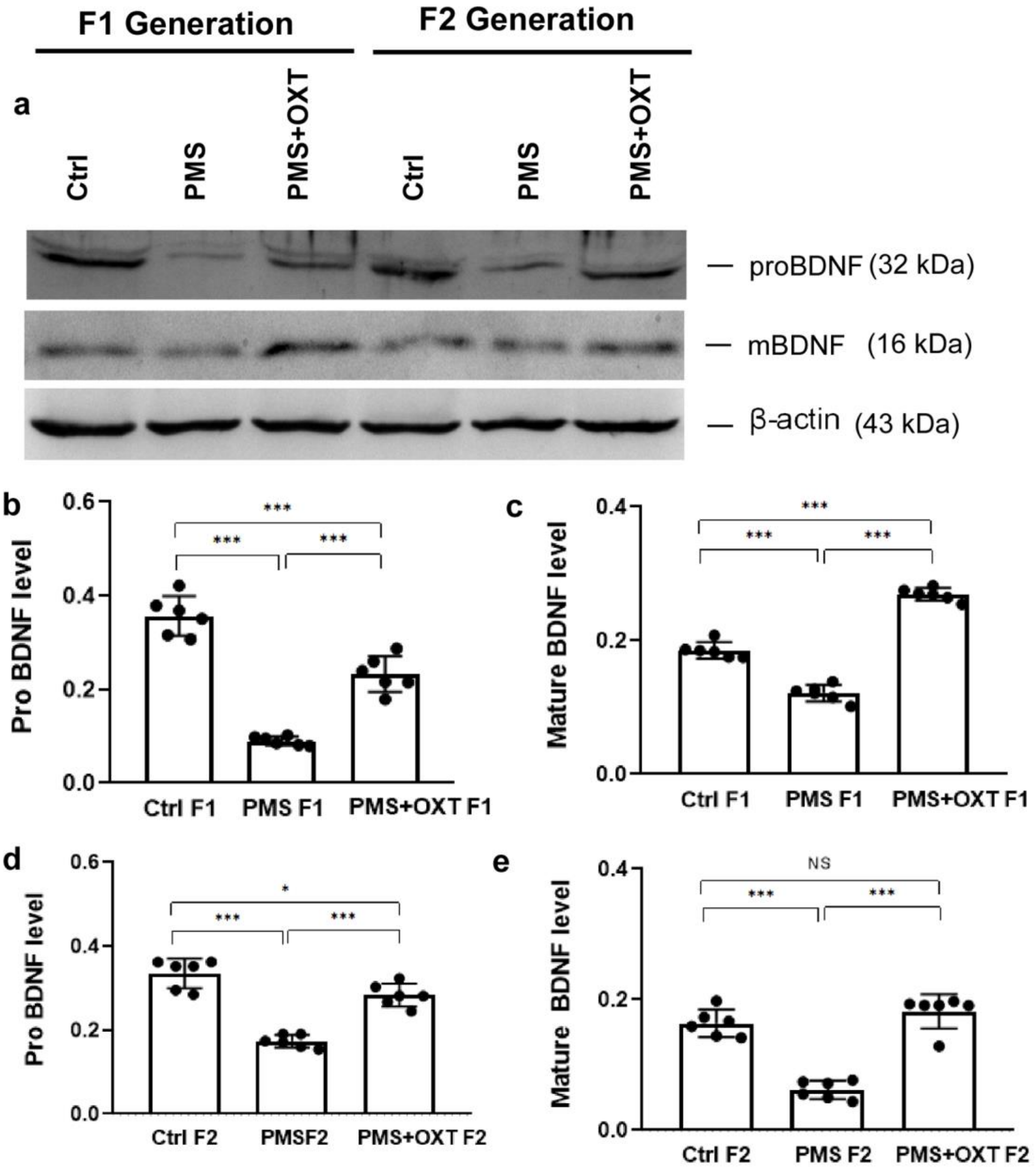

Supplementary Figure 7 Effect of prenatal maternal stress (PMS) alter the level of pro-BDNF and mature BDNF in the F1 and F2 generations. **(a)** Representative western blots showing the expression levels of pro-and mature BDNF in the F1 and F2. The analysis showed that PMS induced effect was similar in pro-BDNF (b, d), mature BDNF (c, e) in F1 and F2 offspring. The level of pro-and mature BDNF was significantly reduced in F1 and F2 offspring from PMS. . Administration of oxytocin minimized the PMS-induced effect, restored the level of pro-and mature BDNF in F1 (PMS+OXT) and F2 (PMS+OXT) offspring Data are represented as mean ± SE, and statistical significance is indicated by **p<0.01; ***p<0.001; NS, not significant.

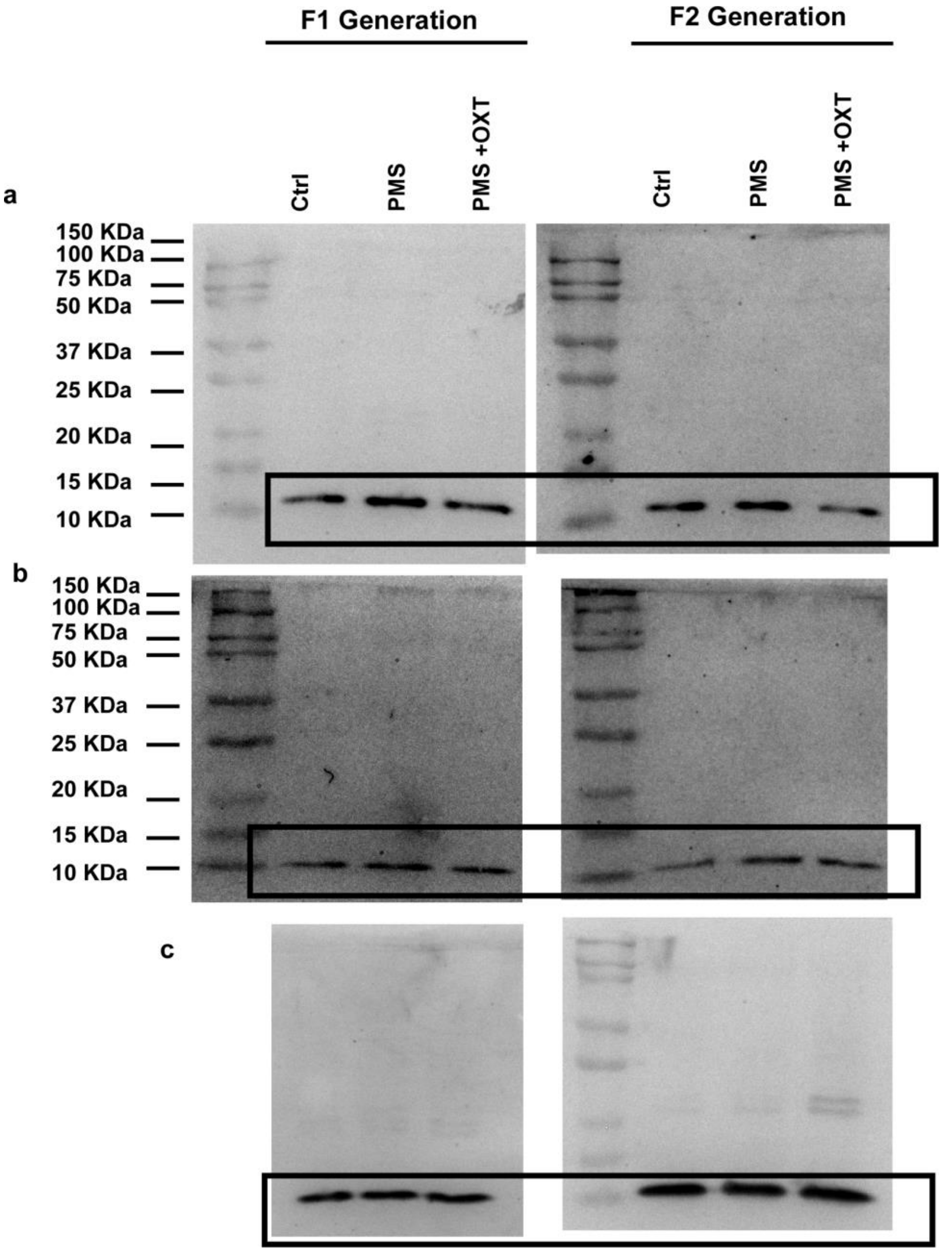

Supplementary Figure 8. Full immunoblot (uncropped) (a) H3K4me2 (first panel), H3K4m3 (second panel) and total H3 (third panel) used for figure 4 in the manuscript. Gray rectangles are the images cropped from each blot that are shown in the manuscript and each lane representing experimental groups [Marker: Pricision Plus Dual Color Standard (Cat# 161-0374) Ctrl: Control; PMS: prenatal maternal stress group; Prenatal maternal stress and treated with oxytocin: PMS+OXY].

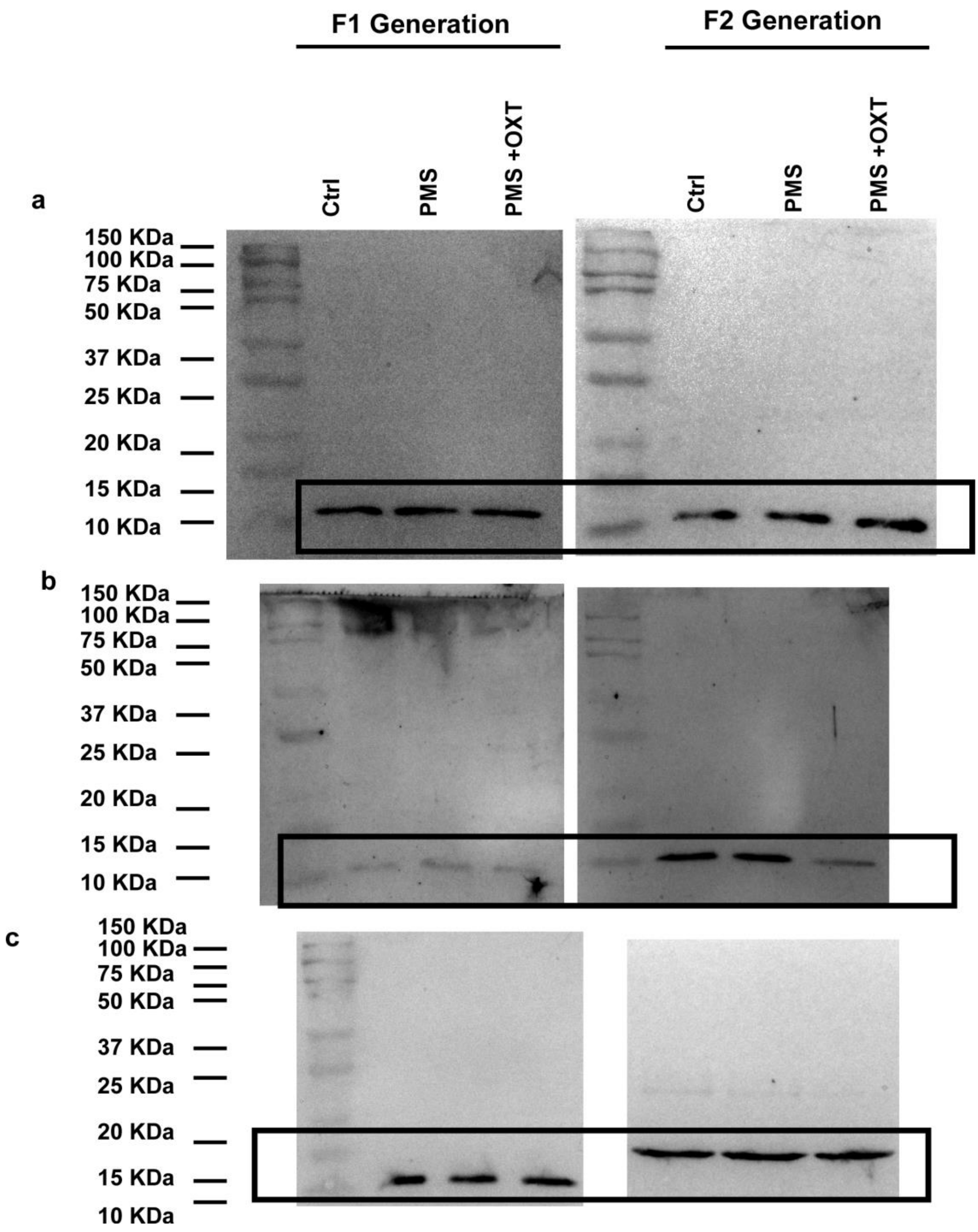

Supplementary Figure 9 (additional replicate). Full immunoblot (uncropped) (a) H3K4me2 (first panel), H3K4m3 (second panel) and total H3 (third panel) used for figure 4 in the manuscript. Gray rectangles are the images cropped from each blot that are shown in the manuscript and each lane representing experimental groups [Marker: Pricision Plus Dual Color Standard (Cat# 161-0374) Ctrl: Control; PMS: prenatal maternal stress group; Prenatal maternal stress and treated with oxytocin: PMS+OXY].

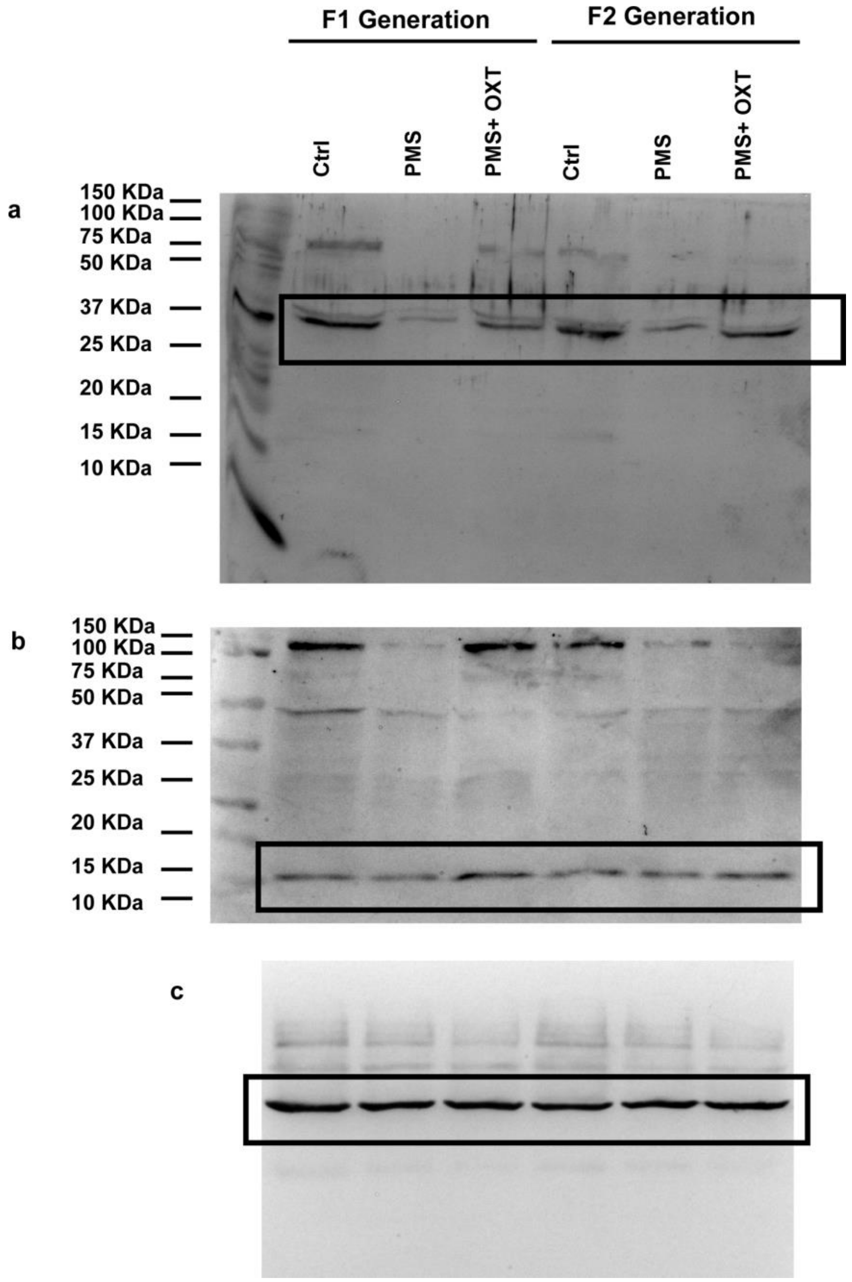

Supplementary Figure 10. Full immunoblot (uncropped) (a) proBDNF (first panel), (b) mature BDNF (second panel) and Beta-actin (third panel) used for figure 8 in the manuscript. Gray rectangles are the images cropped from each blot that are shown in the manuscript and each lane representing experimental groups [Marker: Pricision Plus Dual Color Standard (Cat# 161-0374) Ctrl: Control; PMS: prenatal maternal stress group; Prenatal maternal stress and treated with oxytocin: PMS+OXY].

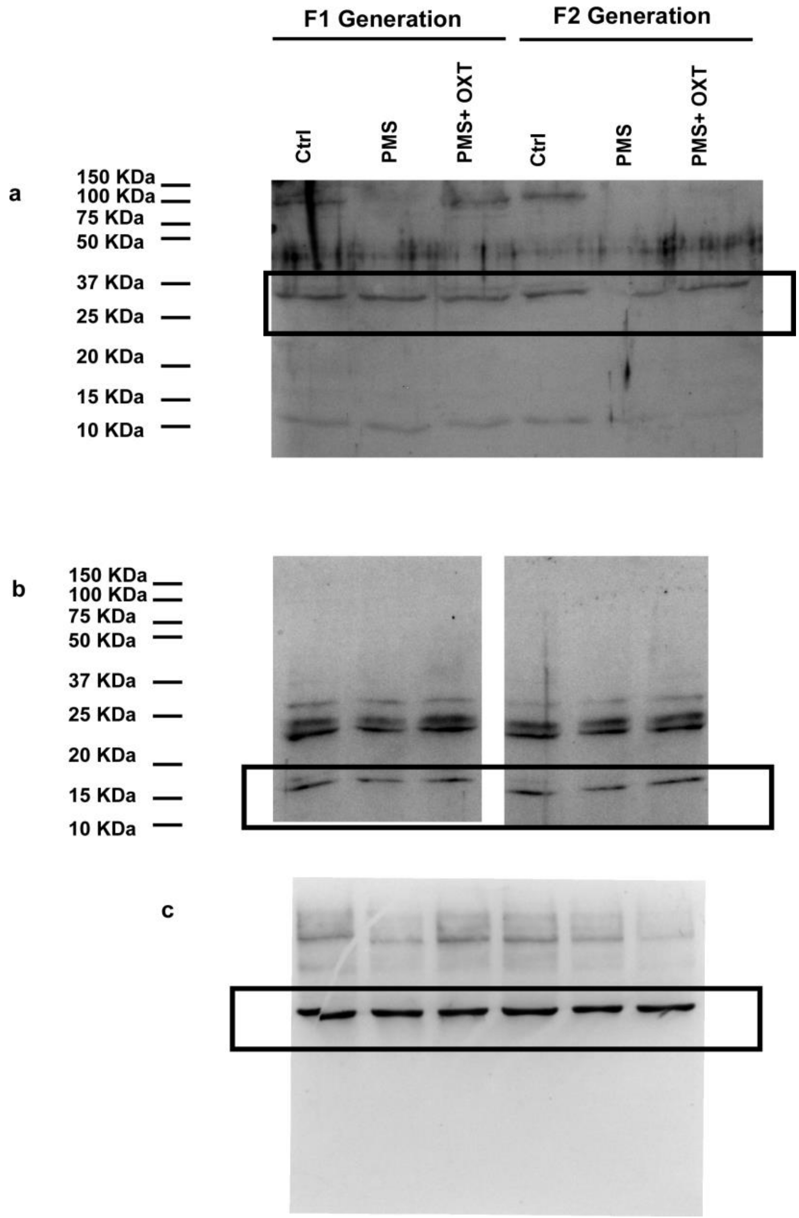

Supplementary Figure 11 (additional replicate). Full immunoblot (uncropped) (a) proBDNF (first panel), (b) mature BDNF (second panel) and Beta-actin (third panel) used for figure 8 in the manuscript. Gray rectangles are the images cropped from each blot that are shown in the manuscript and each lane representing experimental groups [Marker: Pricision Plus Dual Color Standard (Cat# 161-0374) Ctrl: Control; PMS: prenatal maternal stress group; Prenatal maternal stress and treated with oxytocin: PMS+OXY].
